# Comparative transcriptome analysis reveals relationship of three major domesticated varieties of *Auricularia auricula-judae*

**DOI:** 10.1038/s41598-018-36984-y

**Published:** 2019-01-11

**Authors:** Yuhui Zhao, Liang Wang, Dongshan Zhang, Rong Li, Tianyou Cheng, Yibi Zhang, Xueju Liu, Gary Wong, Yuguo Tang, Hui Wang, Shan Gao

**Affiliations:** 1 CAS Key Laboratory of Biomedical & Diagnostic Technology, CAS/Suzhou Institute of Biomedical Engineering and Technology, Suzhou, 215163 China; 2 CAS Key Laboratory of Pathogenic Microbiology and Immunology, Institute of Microbiology, Chinese Academy of Sciences, Beijing, 100101 China; 3 Economic Cooperation Bureau, 11 Mingzhu Road, Jiaohe, Jilin 132500 China; 4 Shanxi Academy of Advanced Research and Innovation, Taiyuan, 030032 China; 5 Shenzhen Key Laboratory of Pathogen and Immunity, Guangdong Key Laboratory for Diagnosis and Treatment of Emerging Infectious Diseases, Shenzhen Third People’s Hospital, Shenzhen, 518112 China; 6 Département de microbiologie-infectiologie et d’immunologie, Université Laval, Québec, QC Canada; 7 Institute of Biomedical Engineering, Old Road Campus, University of Oxford, Oxford, OX3 7DQ UK; 8 Oxford Suzhou Centre for Advanced Research, 388 Ruoshui Road, Suzhou Industrial Park, Jiangsu, 215123 China

## Abstract

*Auricularia auricula-judae* is an edible mushroom and a traditional medicine in China as well as the fourth largest cultivated mushroom species in the world. Here for the first time, we present comparative transcriptome analyses of the fruiting bodies of three morphologically distinguishable *A*. *auricula-judae* cultivated varieties (Wujin, smooth; Banjin, partially wrinkled; and Quanjin, fully wrinkled) collected from Jilin Province, China. Biological triplicates were performed to determine the expression levels of 13,937 unigenes. Among them, only 13 unigenes were annotated to *A*. *auricula-judae*, highlighting the lack of publicly available reference sequences for this economically important species. Principal component analysis (PCA) determined that the gene expression profile of Quanjin was unique when compared to those of Banjin and Wujin. Such relationships were further supported by analyses of annotated and unannotated unigenes, differentially expressed unigenes, gene ontology functions, and the family of peroxidase genes. Using the KEGG database, significant alternations in biological pathways were detected among the three cultivars. This work contributes a large set of *A*. *auricula-judae* sequences to public database, establishes the relationships among major cultivars, and provides molecular guidance for breeding and cultivation.

## Introduction

The *Auricularia auricula-judae* mushroom is commonly known as Jew’s ear, wood ear and jelly ear. The fruiting bodies are routinely consumed as an important edible mushroom in China. Furthermore, *A*. *auricula-judae* is also used as a traditional Chinese medicine with anti-tumor, detoxification, anticoagulant, hypoglycemic, and cholesterol-lowering properties^1–3^. In recent years, *A*. *auricula-judae* has been widely cultivated in large areas of Jilin, Heilongjiang, Henan and Hubei provinces in China, and the yield is ranked fourth in the world for mushroom production^4,5^.

**Figure 1:**
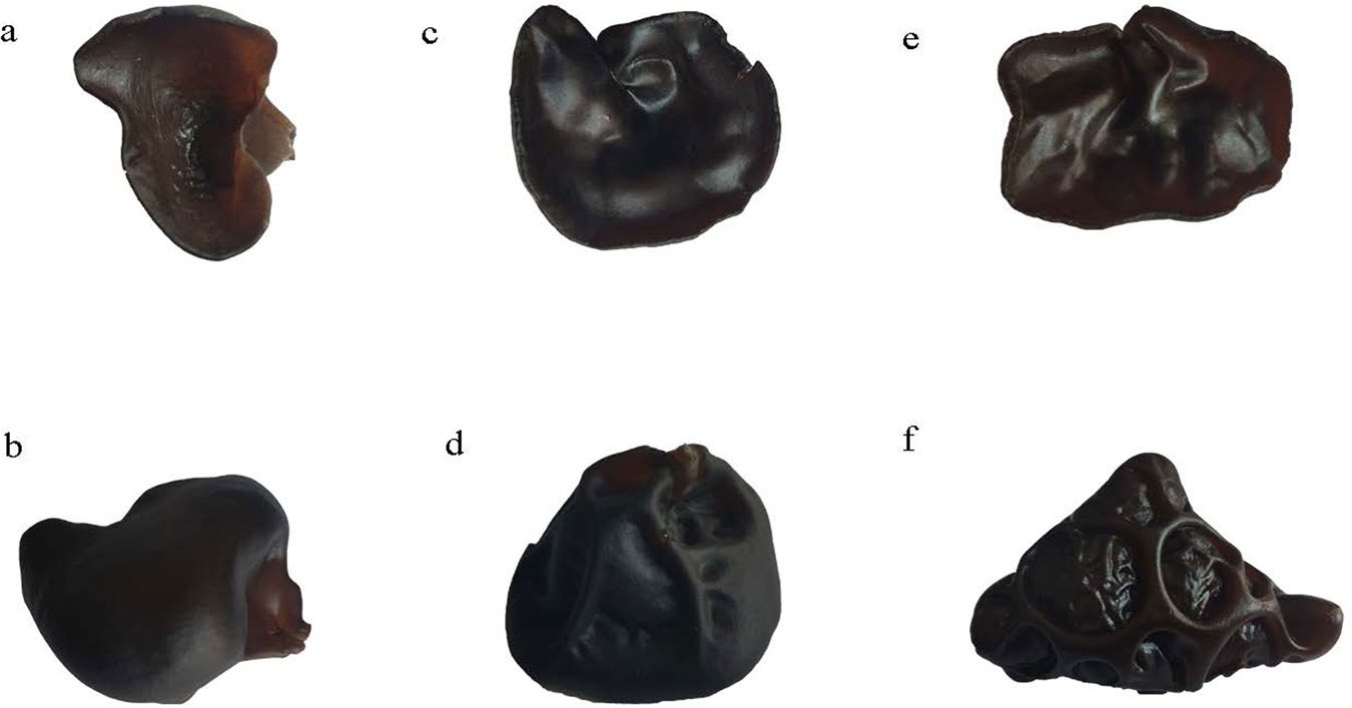
Morphology of the three varieties of *A*. *auricula-judae*. Pictures show the apical and basal sides of the fruiting bodies of Wujin (Panels a and b), Banjin (Panels c and d), and Quanjin (Panels e and f).

Based on the morphological characteristics of fruiting bodies, there are three major *A*. *auricula-judae* varieties in China: Wujin (smooth), Banjin (partially wrinkled), and Quanjin (fully wrinkled) (Fig. 1). The surface wrinkles (also known as veins) have been described as one of the major features of fruiting bodies^6–8^. The number and depth of wrinkles were deployed for cultivar identification purposes in China^7^. The physiological function of the wrinkles is to be determined although possibilities on reflecting nutrition and quality are widely implicated in the food market. All three varieties in China are believed to have been domesticated from wild origins. Quanjin originated from northern China, whereas Wujin and Banjin were more commonly cultivated in southern regions. Despite previous reports of genetic markers using analyses of inter-simple sequence repeat (ISSR), random amplification of polymorphic DNA (RAPD), and sequence related amplified polymorphism (SRAP)^4,5,9–11^, the molecular/genetic bases of differences between the three major Chinese varieties are largely unknown. Molecular studies can provide accurate identification and clarification of wild and domesticated varieties, thus facilitating breeding programs. However, neither the *A*. *auricula-judae* genome nor any transcriptome dataset is publicly available, representing a major barrier for molecular studies.

Next-generation sequencing (NGS) technology is extensively used in life sciences for genome sequencing, transcriptome sequencing, and metagenomics sequencing^12,13^. Comparative transcriptome analysis is an effective way to compare gene expression patterns between different subjects and to provide insights into biological processes^14,15^. Moreover, transcriptome sequencing does not require a reference genome for data analysis^13,16,17^. Many transcriptome studies have been performed for mushroom species, including *Lentius edodes*^18,19^, *Ganoderma lucidum*^20^, *Agrocybe aegerita*^21^, *Auricularia polytricha*^14^, *Pleurotus eryngii subsp*. *Tuoliensis*^15^ and *Cordyceps militaris*^22^.

To determine the molecular relationships of the three major *A*. *auricula-judae* varieties in China, fruiting bodies from three cultivated strains (each represents a major variety) were examined for gene expression profiles using transcriptome sequencing: 13,937 unigenes were identified, and 12,813 were annotated. Principal component analysis (PCA) revealed that all three varieties had distinguishable gene expression profiles, but Quanjin was unique relative to the other two varieties. Further analysis of differentially expressed unigenes confirmed the PCA result. Such a molecular clarification of the major domesticated varieties provided directional information for developing breeding strategies. In addition, 1,124 unigenes did not display significant homology to any known genes, providing valuable information for further investigations into the biochemical and pharmaceutical properties of *A*. *auricula-judae*.

## Results

### *De novo* assembly of the *A. auricula-judae* transcriptome

**Figure 2:**
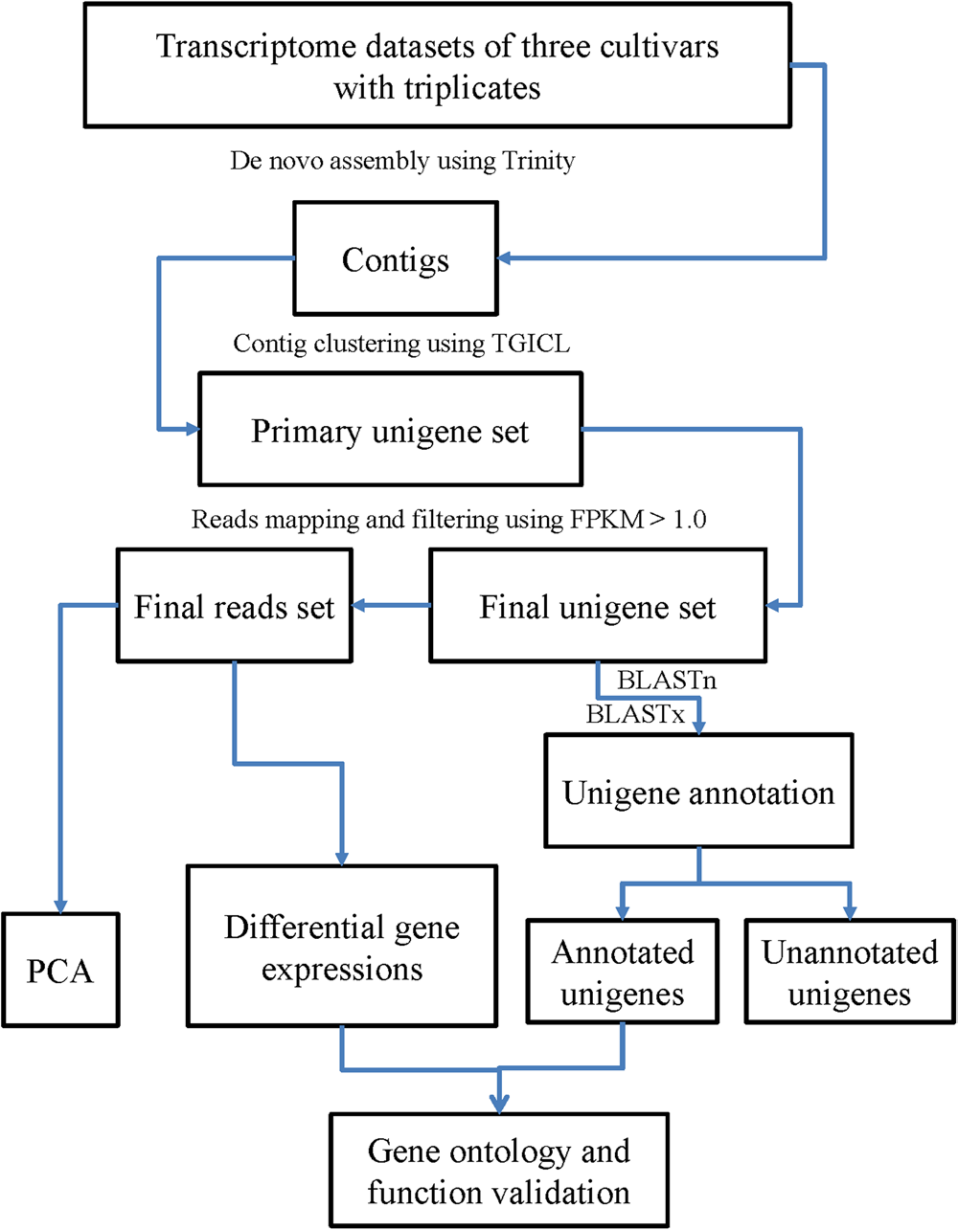
Analytical framework of the *A*. *auricula-judae* transcriptome. Raw reads of each of the three varieties were filtered and then *de novo* assembled into contigs by Trinity. Unigenes were obtained by clustering the contigs with TGICL. FPKM values were calculated for each of the unigenes, and only unigenes with FPKM >1.0 in any of the three varieties were used for further analyses and annotation against the public databases.

Each of the individual RNA samples from Quanjin (n = 3), Banjin (n = 3), and Wujin (n = 3) (Fig. 1) were used to construct cDNA libraries for sequencing on an Illumina HiSeq2000 system. After removal of low quality reads, 69,814,486 reads, 62,918,023 reads, and 69,589,663 reads were obtained for Quanjin, Banjin, and Wujin, respectively (Supplementary Table S1). These high-quality reads were assembled *de novo* using Trinity^23^, and 33,316 contigs, 29,523 contigs, and 33,682 contigs were generated for Quanjin, Banjin and Wujin, respectively. To obtain a non-redundant library of consensus sequences, contigs of the three varieties were merged using TGICL^24^. Primary unigenes (54,320) were obtained containing 20,129 distinct clusters and 34,191 distinct singletons. The sequencing reads were further processed in edgeR^25^ to obtain FPKM (Fragments Per Kilobase of transcript per Million mapped fragments) values for each primary unigene in each of the biological samples (n = 9). For each variety, median FPKM value of the three replicates was used to represent the expression level. Only unigenes with a FPKM > 1.0 in any of the three varieties were collected for further analysis (Fig. 2). Ultimately, a final unigene library containing 13,937 unigenes (length range 201–30,448 bp) were established. The average and N50 lengths of unigenes were 2,594 and 3,521 bp, respectively (Supplementary Table S2).

### Annotation of unigenes

**Figure 3:**
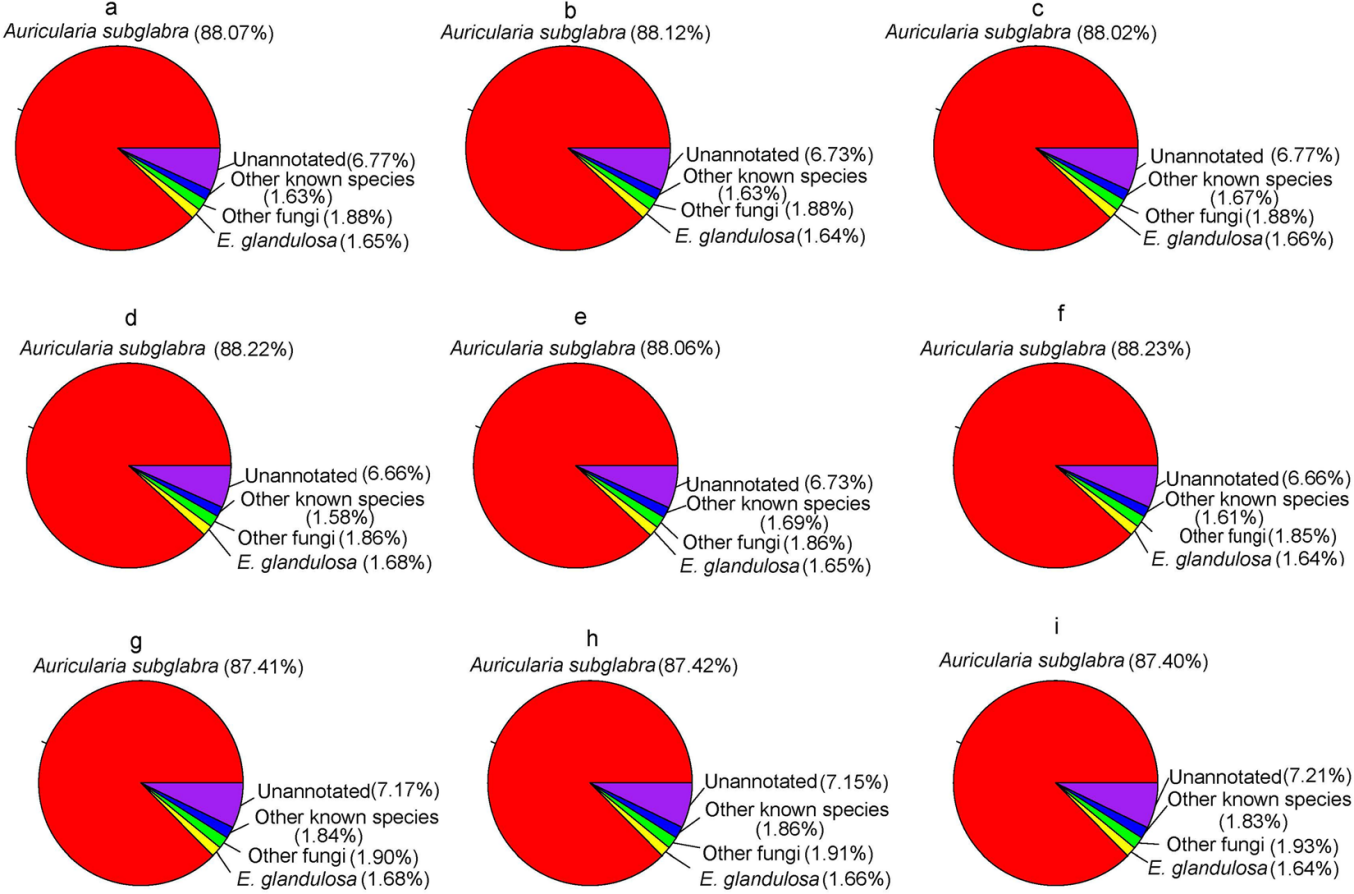
Summary of unigene annotation. Unigenes were divided into five major annotation groups (percentage >1% of total) and the proportion of each group is shown for each of the individual samples of Wujin (n = 3, Panels a–c), Banjin (n = 3, Panels d–f), and Quanjin (n = 3, Panels g–i).

To identify if the unigenes were homologues of any known genes, a BLASTn search against the NCBI non-redundant nucleotide (nt) databases was performed and 12,011 unigenes were annotated. These unannotated unigenes were then searched against the Swiss-Prot and NCBI non-redundant protein (nr) databases using BLASTp with an E-value cutoff of 1e-4. Among the remaining 1,926 unigenes, 802 had significant similarities to known proteins. In total, 12,813 unigenes were annotated to known genes/proteins (Supplementary Dataset S1–S3). Only 0.0988% (median, n = 3), 0.101% (median, n = 3) and 0.101% (median, n = 3) of the unigenes were annotated to *A*. *auricula-judae* from varieties of Quanjin, Banjin and Wujin, respectively (Supplementary Table S3). Instead, the majority of unigenes were annotated to *Auricularia subglabra* (Fig. 3), highlighting lack of *A*. *auricula-judae* reference sequences in the public databases. In total, there were 1,124 unannotated unigenes among the nine samples (Supplementary Dataset S4).

### Transcriptomic relationships between the three *A*. *auricula-judae* varieties

**Figure 4:**
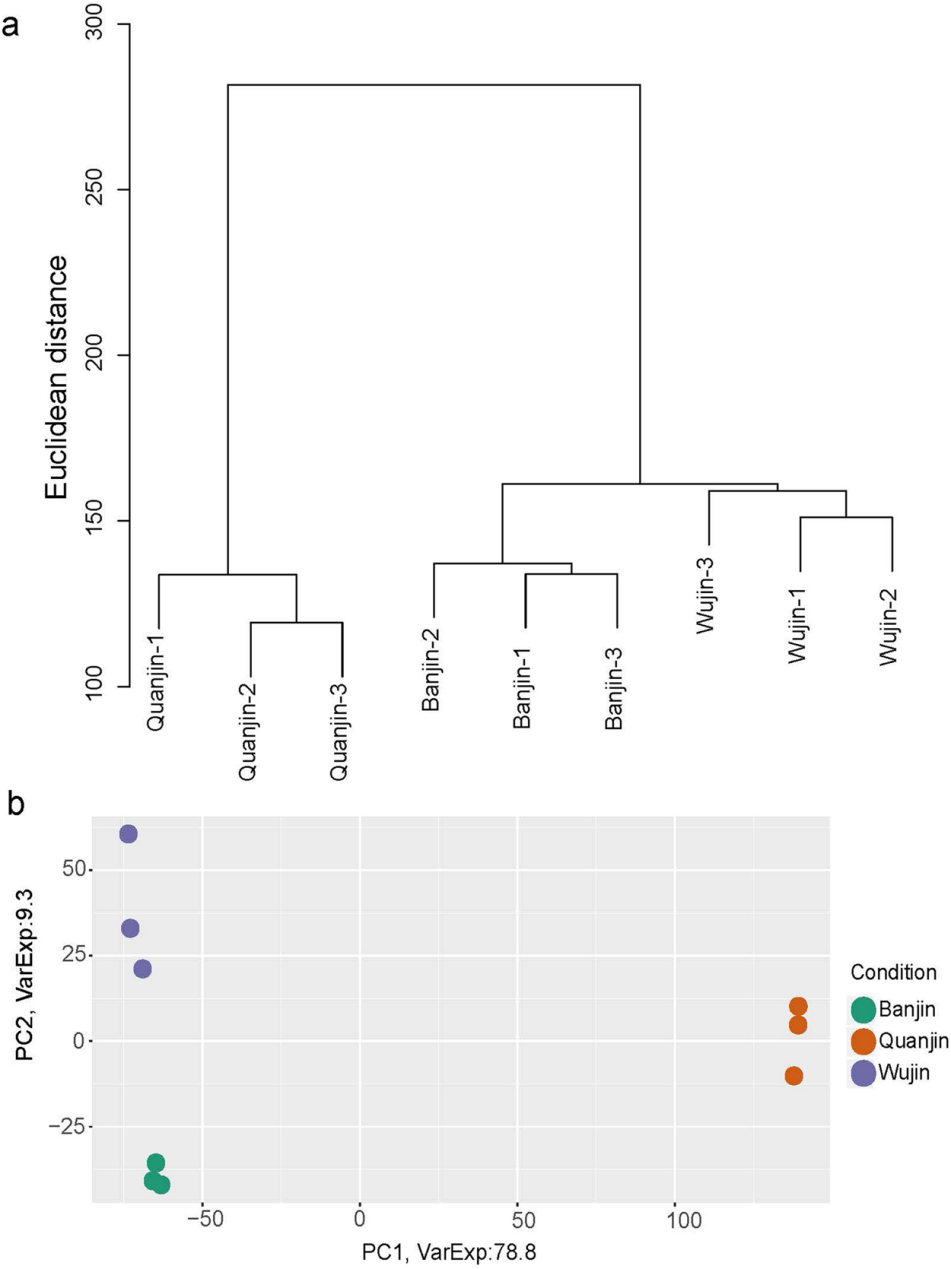
Gene expression relationships among the three *A*. *auricula-judae* varieties. (Panel a) Raw reads counts of unigenes in each of the samples were transformed into regularized log–transformed data by rlogTransformation. Then, the distance between samples was calculated and displayed in a hierarchical clustering tree by hclust. The X-axis labels samples, and the Y-axis represents the Euclidean distance between samples. (Panel b) The top two principal components (X-axis, PC1; Y-axis, PC2) deciphered 88.1% of all difference among the three varieties. Banjin and Wujin were similar to each other in PC1 but showed a greater difference in PC2 when compared to Quanjin.

To determine the gene expression pattern relationships among the three varieties of *A*. *auricula-judae*, a cluster analysis using final reads set (Fig. 2) was performed. A Euclidean distance tree revealed that all three biological triplets clustered together in all varieties (Fig. 4a), confirming good sample quality. Quanjin (n = 3) was clearly isolated from Banjin (n = 3) and Wujin (n = 3), indicating an overall difference between their transcriptomic characteristics (Fig. 4a). PCA was also performed to confirm the relationships between the three varieties (Fig. 4b). The PCA revealed that Quanjin (n = 3) was clearly separated from Banjin (n = 3) and Wujin (n = 3) at both PC1 and PC2, whereas Banjin and Wujin appeared distinguishable only at PC2. At PC2, however, Quanjin (n = 3) was flanked by Banjin (n = 3) and Wujin (n = 3), indicating that Quanjin had more similarities to Banjin and Wujin than those shared between the latter two varieties in certain aspects (Fig. 4b). Therefore, the three mushroom varieties had a triangular relationship in their gene expression patterns. In addition to the between-variety relationships, the PCA results also revealed a visible difference in between-individual variations among the three varieties. Banjin displayed the least variation, whereas Wujin was the most variable, though the between-individual variations were mainly evident at PC2 (Fig. 4b).

**Figure 5:**
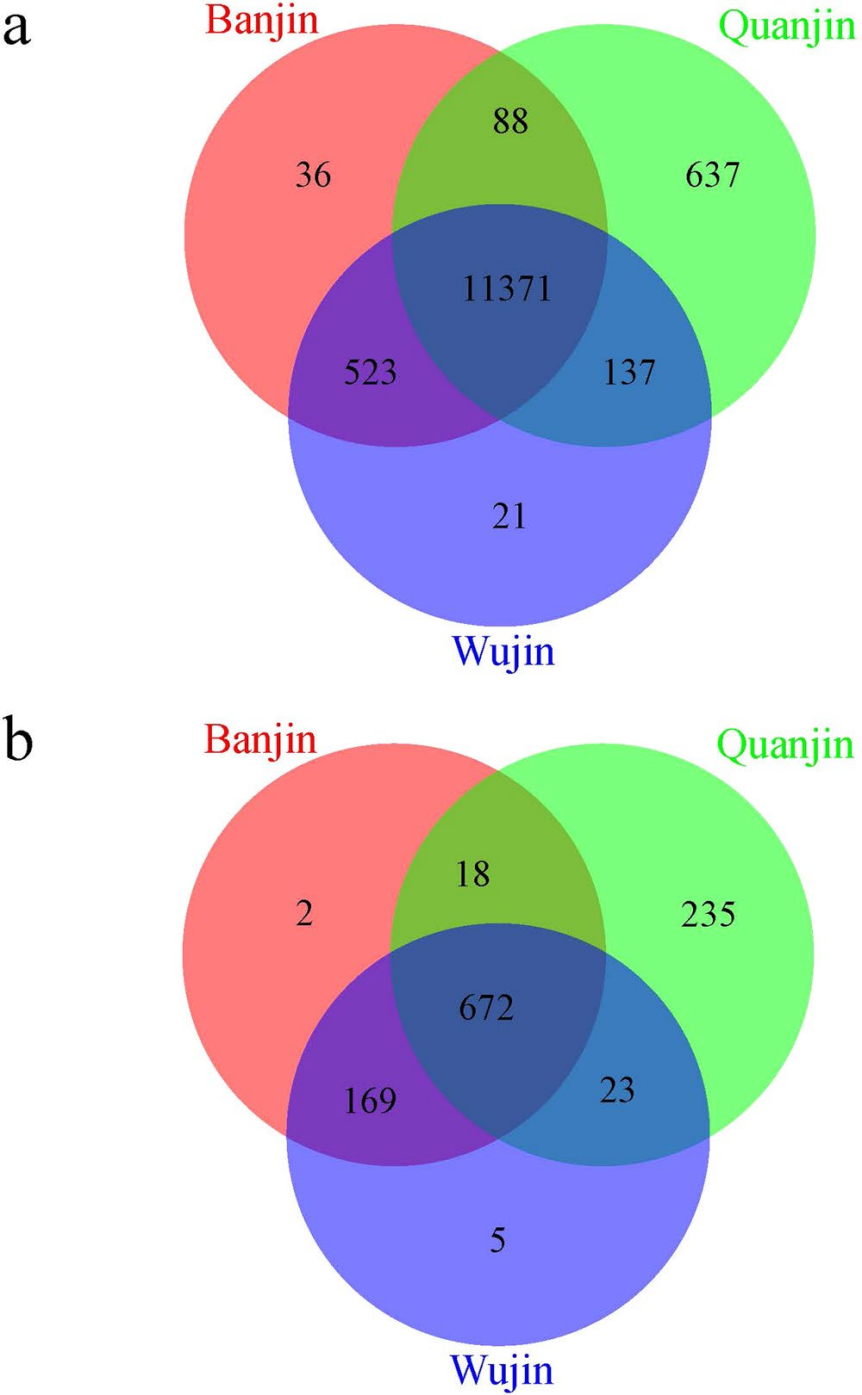
Unigene expression. The numbers of unigenes detected in the three *A*. *auricula-judae* varieties are displayed in Venn charts. (Panel a) Expression of unigenes annotated to *Auricularia subglabra*, *E*. *glandulosa*, and other species (Fig. 3). (Panel b) Expressions of unannotated unigenes (Fig. 3).

Among the 12,813 unigenes annotated to known genes/proteins, the expression of 11,371 (88.7%) unigenes was shared in all three varieties (Fig. 5a). Quanjin uniquely expressed 637 annotated unigenes, whereas only 36 and 21 unigenes were uniquely detected in Banjin and Wujin, respectively. However, the latter two shared an intersection of 523 annotated unigenes whose expression was not detected in Quanjin (Fig. 5a). Between these sets of 637 Quanjin unique unigenes and 523 Banjin-Wujin shared unigenes, 141 pairs of unigenes had nucleotide sequence similarities (determined by local BLASTn) and 57 pairs of unigenes had amino acid sequence similarities (determined by local BLASTx), suggesting that they may perform similar functions but have polymorphisms in the sequences. Taken together, the above results indicated that Banjin and Wujin transcriptomes are closely related to each other, while they were distant to Quanjin in the gene expression landscape.

**Figure 6:**
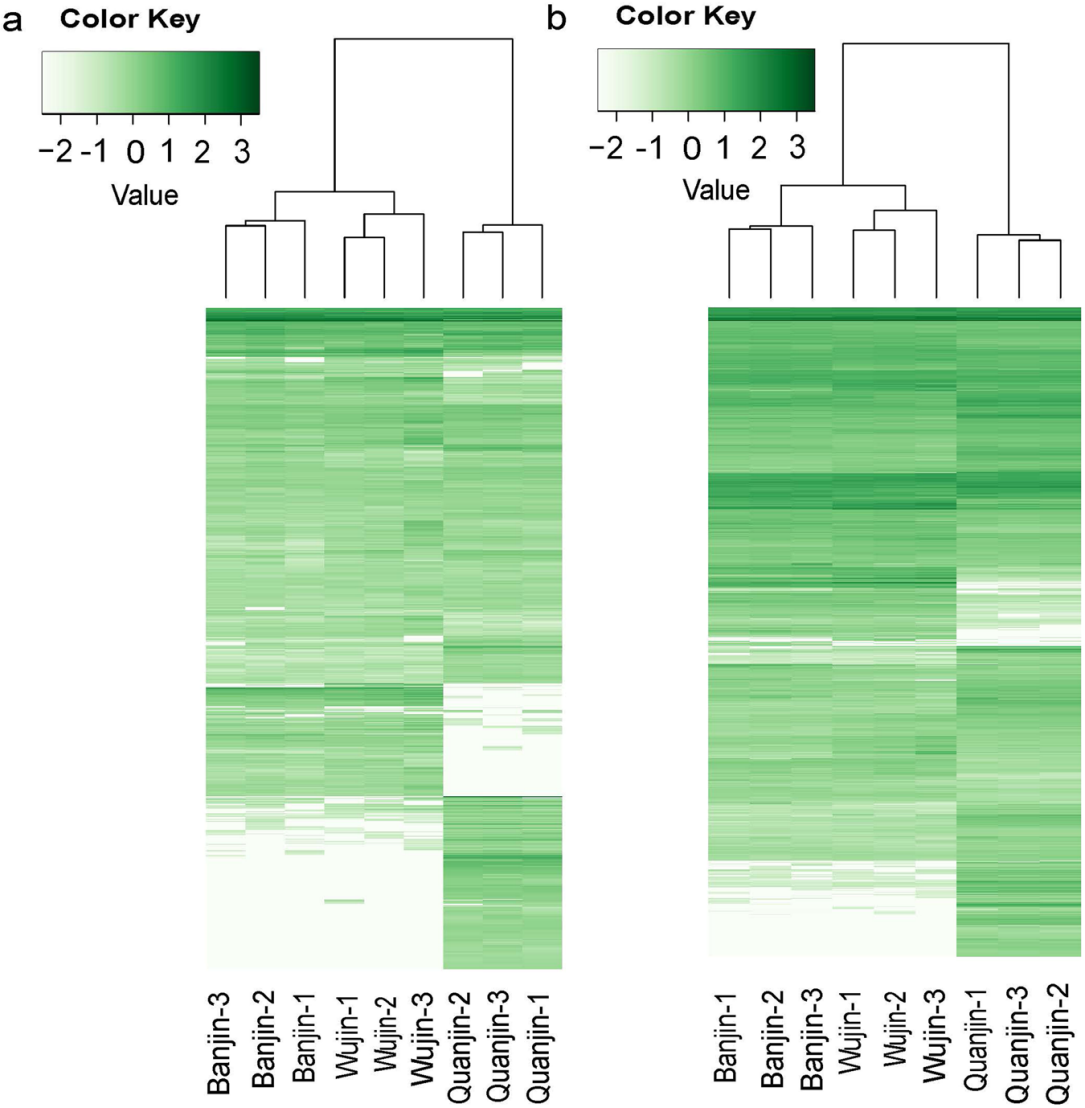
Expression profiles of novel and differentially expressed unigenes. The FPKM of all *A*. *auricula-judae* unigenes in nine samples were scaled together and transformed into Z-scores ((x-mean)/(s.d.)) using an in-house Perl program and are represented in a clustering heat map by Heatmap 2. The Z-scores are color coded as shown in the color bar. (Panel a) Expression profile of novel unigenes. (Panel b) Expression profile of differentially expressed unigenes.

Among the 1,124 unannotated unigenes, 672 (59.8%) were shared by all of the three *A*. *auricula-judae* varieties. Quanjin uniquely expressed 235 novel unigenes, whereas 169 novel unigenes were only detected in the intersection between Banjin and Wujin (Fig. 5b). Thirty pairs of homologues were detected in these two sets of novel unigenes by local BLAST. The expression levels of these novel unigenes in all nine samples are displayed in a clustering heat map using the FKPM values (Fig. 6a). The expression patterns of the novel unigenes (Fig. 6a) conformed to the whole transcriptome characteristics and supported that Quanjin was isolated from Banjin and Wujin (Fig. 4).

### Differentially expressed unigenes

Differentially expressed unigenes (DEGs) were determined for each of the two variety pairs using EdgeR with a cutoff threshold of *P* < 0.05. There were 2,861 DEGs in Banjin *vs*. Wujin, 5,992 DEGs in Quanjin *vs*. Banjin, and 6,549 DEGs in Quanjin *vs*. Wujin (Supplementary Table S4). An expression clustering heat map of all identified DEGs was produced for all nine samples using the FKPM values (Fig. 6b). The DEG clustering results also conformed to the whole transcriptome clarification that Quanjin was more divergent compared to the other two *A*. *auricula-judae* varieties (Fig. 4).

### Gene ontology (GO) annotation and cluster analysis of function groups

**Figure 7:**
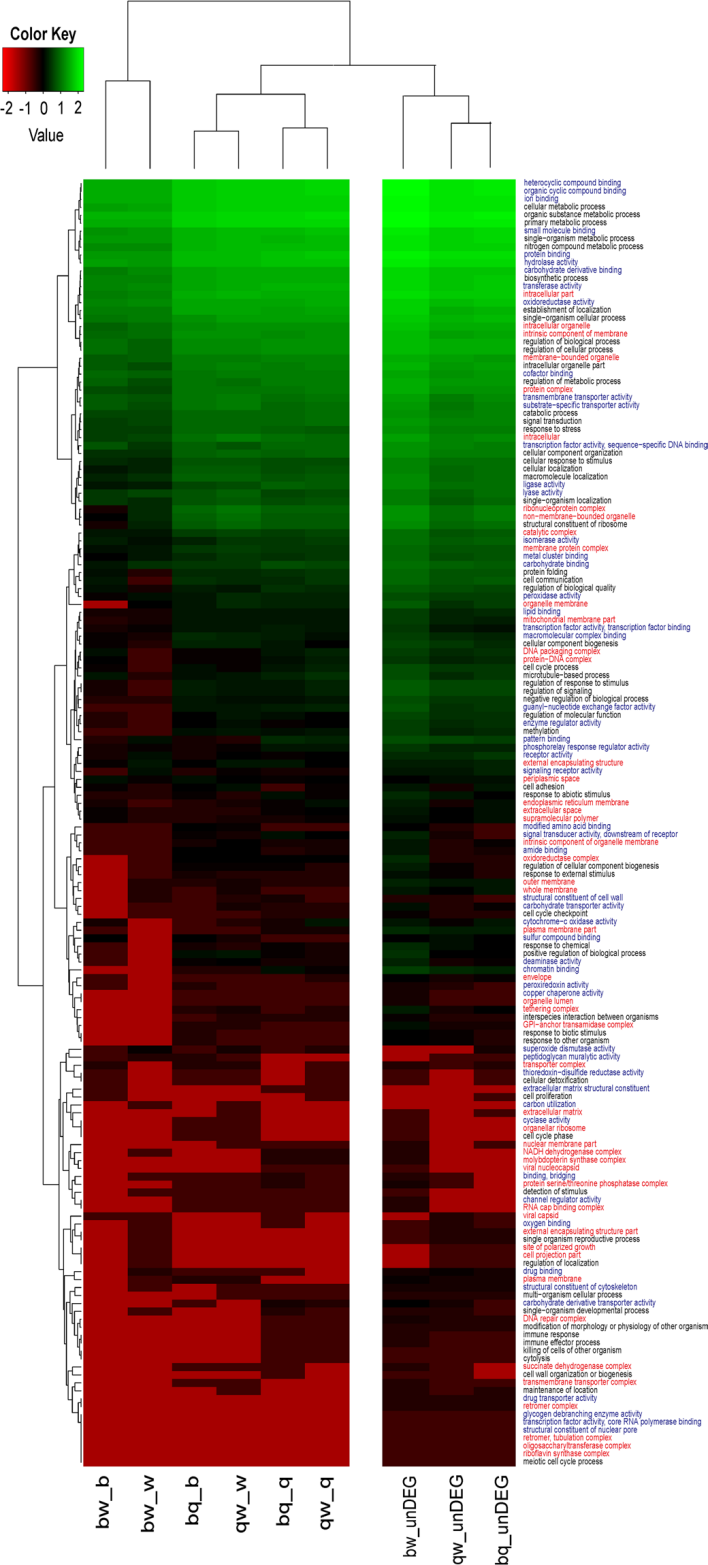
GO of DEGs and unDEGs. The numbers of unigenes in each GO term were collected for DEGs (*P* < 0.05) and unDEGs, respectively, for each pairwise comparison among the three varieties. All of the unigene numbers were normalized into Z-scores ((x-mean)/(s.d.)) using an in-house Perl program and are represented in a clustering heat map by Heatmap 2. The Z-scores are color coded as shown in the color bar. Color word labeling was used to represent different classes of GO categories of molecular function (blue), cellular component (red), and biological process (black).

The 12,813 BLAST-annotated *A*. *auricula-judae* unigenes were further annotated to 162 GO functions. Among them, 55 GO functions belonged to the category of molecular function, 50 belonged to cellular component, and 57 belonged to biological process (Fig. 7, Supplementary Dataset S5). Based on the number of unigenes in each GO function detected in each of the three varieties, a GO function clustering analysis was performed to summarize the relationships among the three varieties at the GO function level. The numbers of unigenes were transformed together into Z-scores and displayed separately as DEGs and un-DEGs (Fig. 7). Again, Banjin and Wujin displayed greater similarities than those of Banjin *vs*. Quanjin and Wujin *vs*. Quanjin. This is shown in Fig. 7 in two aspects as the greatest number of un-DEGs and the lowest numbers of DEGs between Banjin and Wujin (Fig. 7 and Supplementary Dataset S5), indicating similarities between the two varieties. There was further supporting evidence from the DEGs section, where the columns of up-regulated Quanjin unigenes clustered together (bq-q and qw-q, Fig. 7) and was isolated from the cluster containing columns of down-regulated Quanjin unigenes (bq-b and qw-w, Fig. 7), indicating similar patterns for the difference between Qianjin *vs*. Banjin and Qianjin *vs*. Wujin. Based on these results, the functional characteristics of Quanjin transcriptome were indeed unique compared to those of Banjin and Wujin.

### KEGG pathway enrichment associated with surface wrinkle

**Figure 8:**
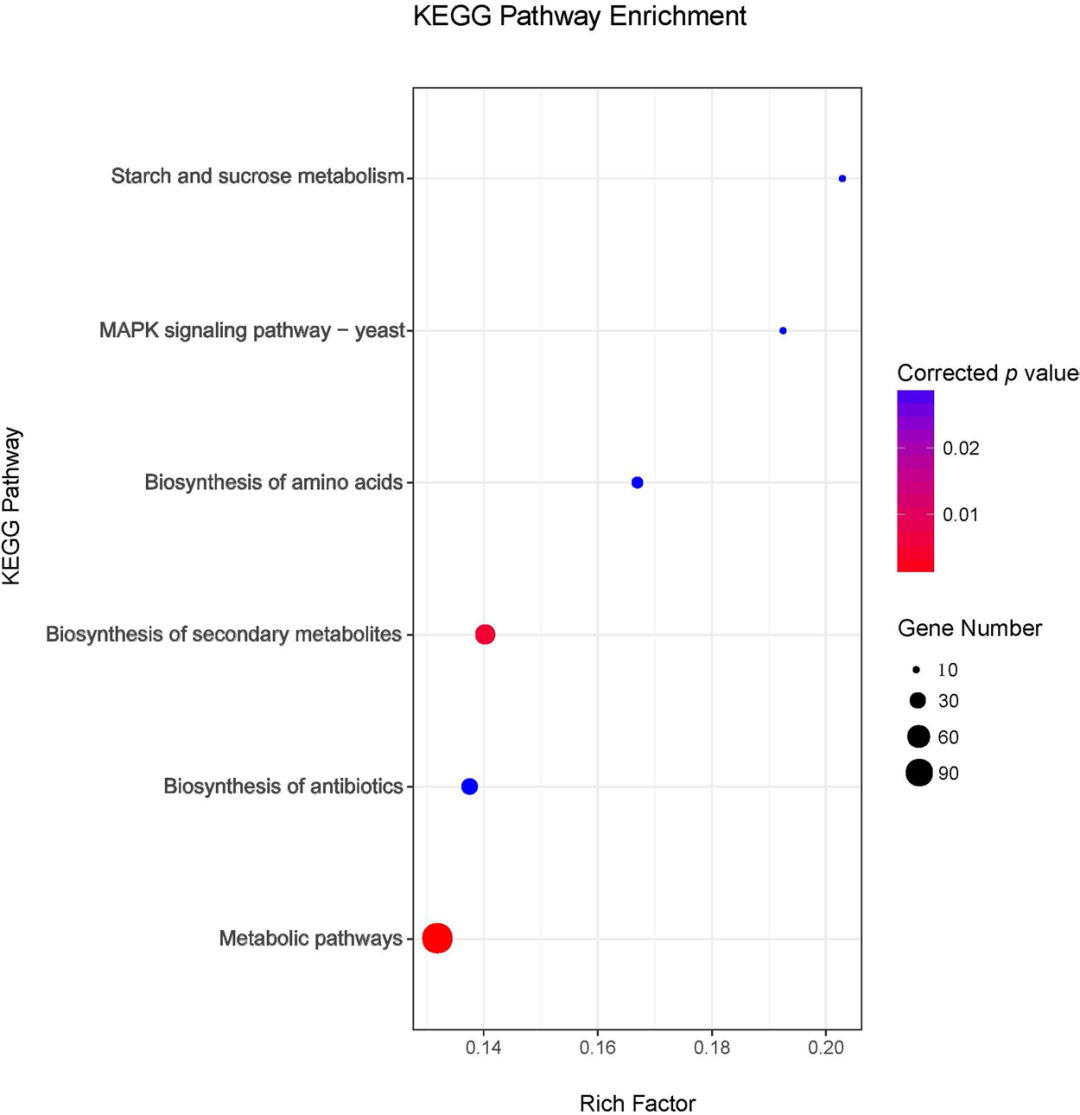
KEGG pathways associated with fruiting body wrinkle Scatter plot of KEGG pathways enriched for unigenes that were up- or down-regulated by the wrinkle morphology. The X-axis displays the Rich Factor which is the ratio of numbers of the unigenes in a pathway term to all gene numbers annotated in the same pathway term. The size of bubble represents the number of unigenes and the color gradient indicates the corrected *P* value by Benjamini-Hochberg FDR method. Only pathways with corrected *P* < 0.05 were shown.

983 unigenes were extracted for wrinkle down-regulation (FPKM: Wujin > Banjin > Quanjin) and 988 unigenes were extracted for wrinkle up-regulation (FPKM: Wujin < Banjin < Quanjin). The total of 1971 unigenes were pooled and determined for significant enrichments (with corrected *P* < 0.05, by Benjamini-Hochberg FDR method) in six KEGG pathways, such as starch and sucrose metabolism (Rich Factor, number of enriched genes/number of total genes in the pathway, RF = 0.20), MAPK signaling pathway-yeast (RF = 0.19), biosynthesis of amino acids (RF = 0.17), biosynthesis of secondary metabolites (RF = 0.14), biosynthesis of antibiotics (RF = 0.14) and metabolic pathways (RF = 0.13) (Fig. 8, Supplementary Dataset S6). To our knowledge, this is the first description of biological functions possibly associated with fruiting body wrinkles, a major morphological feature of *Auricularia* mushrooms^6–8^.

### Expressions of peroxidase-like unigenes

**Figure 9:**
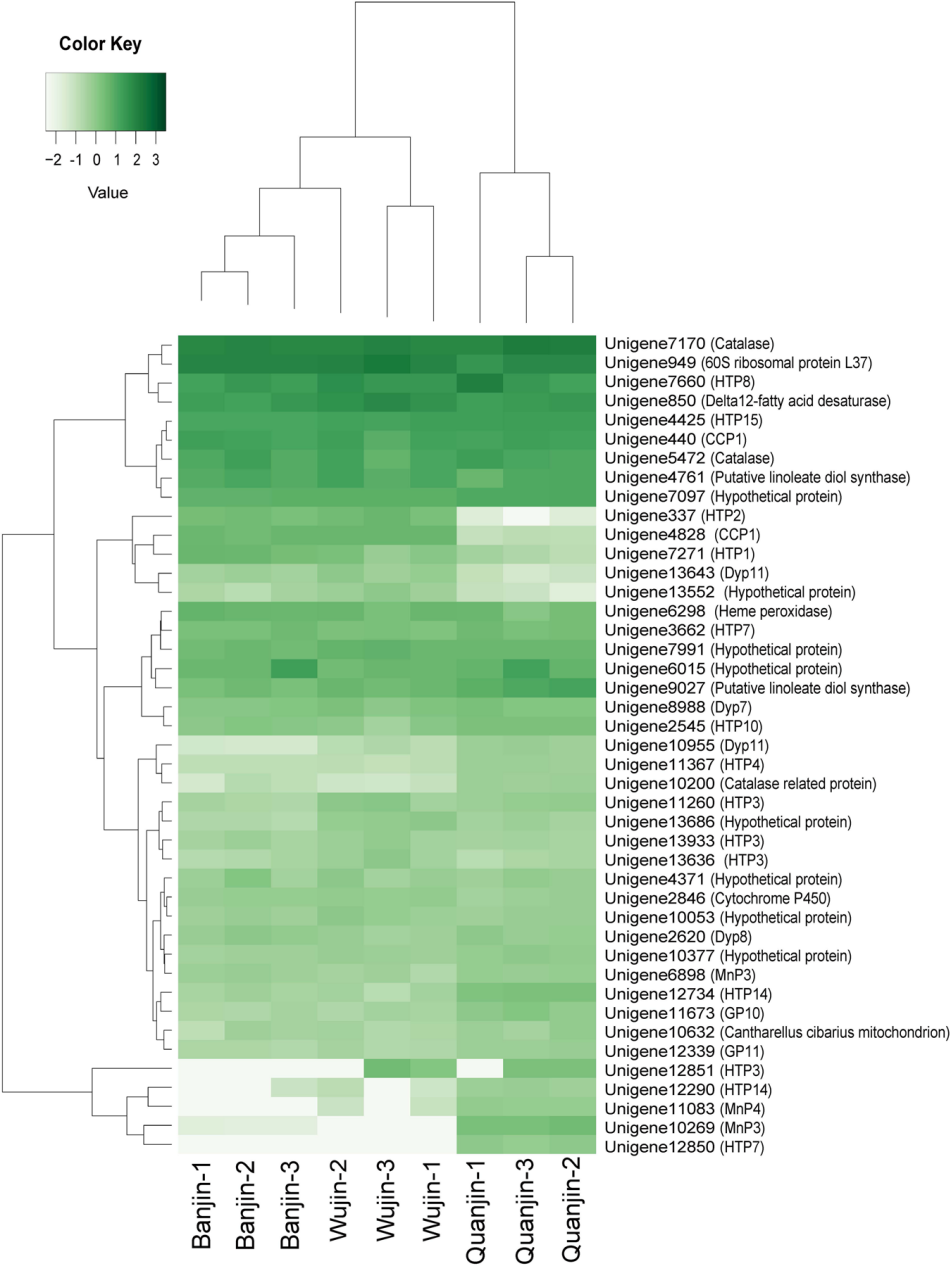
Expression profiles of peroxidase-related unigenes. The Z-scores ((x-mean)/(s.d.)) of FPKM values of *A*. *auricula-judae* peroxidase-related unigenes were extracted from the Z-scores of all unigenes and are represented in a clustering heat map by Heatmap 2. The Z-scores are color coded as shown in the color bar. Forty-three unigenes were functionally annotated to peroxidase by GO and were labelled using the unigene ID followed by the BLASTn/x annotations (Supplementary Dataset S7).

To further validate the relationship of functional characteristics among the three *A*. *auricula-judae* varieties, we examined the expression patterns of peroxidase-like unigenes in all nine samples. In total, 43 unigenes displayed homologies to known peroxidases (Supplementary Dataset S7), including 14 heme-thiolate peroxidase (HTP)-like unigenes, four DyP-type peroxidase-like unigenes, and three manganese peroxidase (MnP)-like unigenes (Supplementary Figure S1). Figure 9 shows a clustering heat map based on the FKPM values. Consistent with previous results (Figs 4, 6 and 7), Quanjin (n = 3) was isolated from Banjin (n = 3) and Wujin (n = 3) samples, confirming that the expression profiles of peroxidase-like genes in Quanjin were unique when compared to those of Banjin and Wujin (Fig. 9). However, from the peroxidase expression profiles, clustering analysis could not sufficiently separate Banjin and Wujin, mainly due to a large between-individual variation in Wujin (Fig. 9, Wujin-2). This result conformed to the PCA results that the greatest between-individual variation was observed in Wujin when all unigenes were used (Fig. 4b).

### Real time PCR validation of selected peroxidase-like unigene transcriptions

To validate the transcriptome results, 16 peroxidase-like unigenes (Supplementary Figure S1) were selected for RT-qPCR analysis. Firstly, Sanger sequencing of the RT-qPCR products (Supplementary Dataset S8) confirmed genuine amplifications of all of the 16 peroxidase-like transcripts tested (Supplementary Dataset S9). Secondly, the ΔCt values had negative correlations (*P* < 0.05) to the FKPM values in all three cultivars (Supplementary Table S5), showing compatibility of expression levels determined by the two methods. Interestingly, transcriptions of *A*. *aj DyP1* (JQ650250)^26^ were detected by RT-qPCR with relatively high ΔCt values (Supplementary Table S6) in all three cultivars although its homologue was not included in our final unigene set of FPKM > 1.0. Further investigation using contigs detected signals of two non-overlapping JQ650250 homologues with low FPKM values (Supplementary Table S6), emphasizing that the final unigene set excluded lowly expressed *A*. *auricula-judae* genes.

## Discussion

In 2012, the genome of *Auricularia subglabra* was sequenced and annotated^27^. The transcriptome of *A*. *polytricha* was sequenced using the Illumina RNA-seq protocol, and a gene expression profile was reported in 2014^14^. Recent studies of *A*. *auricula-judae*, an important edible mushroom in China, however, are largely limited in classification, phylogenetic analysis, biodiversity, and conservation issues using ISSR, RAPD, and SRAP^5,9–11^. Knowledge of the genomics and transcriptomics of *A*. *auricula-judae* is needed to facilitate research activities and agricultural developments. To address a fundamental question concerning the gene expression characteristics of three major Chinese domesticated *A*. *auricula-judae* varieties, we applied comparative transcriptome analysis to the fruiting bodies of Quanjin, Banjin and Wujin. A clear relationship was determined that Quanjin displayed major differences in gene expression compared to Banjin and Wujin (Fig. 4b, PC1), conforming to a previous report that *A*. *auricula-judae* strains with different fruiting body wrinkle characteristics belonged to two major types based on SRAP classification^28^. This observation was further supported by expression patterns of both annotated unigenes (Fig. 7) and unannotated novel unigenes (Fig. 6a), as well as DEGs and unDEGs (Fig. 7). Functional validations also supported the conclusion by GO function categorization (Fig. 7) and the expression of peroxidase-like genes (Fig. 9). This conclusion supports the notion that Wujin and Banjin may have a genetic relationship or common ancestor from southern China, but that Quanjin had its own origin from northern China. Genomic sequence data would be needed to reveal the full picture of genetic relationship^29^. Encouragingly, during reviewing period of this manuscript, genomes of 90 mushroom species were published^30^. From a genome of *A*. *heimuer*, 16,244 putative genes were predicted^31^, comparable with the number of unigenes reported in this study.

The dye de-colourizing peroxidase (DyPs) are a family of heme peroxidases that are considered possible resources for the bioremediation of lignin. DyPs have been isolated from *A*. *auricula-judae* with unique sequence and enzymatic features^26^. We detected the expression of four previously unreported DyP-like unigenes based on their sequence homology to *Auricularia subglabra* DyPs (Supplementary Figure S1b). Expression of the previously reported *A*. *auricula-judae* DyP (*A*. *aj-DyP1* derived from Strain DSMZ 11326, NCBI accession number JQ650250, Supplementary Figure S1b), however, was not detected by the transcriptome final unigene set (Supplementary Datasets S1–S3) but detected by RT-qPCR (Supplementary Table S6). Further investigation on the assembly result revealed two non-overlapping contigs that displayed more than 99% identity to JQ650250 but had low expression levels (FPKM < 0.3), emphasizing that our final unigene set excluded lowly expressed genes due to the filtration of FPKM > 1.0. The low expression levels of *A*. *aj-DyP1* may be due to possible genotypic differences between the three Chinese varieties we used and Strain DSMZ 11326 from which *A*. *aj-DyP1* was discovered. It may also be due to phenotypic factors that can be affected by environmental and/or cultivation conditions. Nevertheless, the diversities of *A*. *aj-DyPs* (Fig. 9, Supplementary Fig. S1b) are an excellent example of the high degrees of variation in *A*. *auricular-judae* gene expression. Clarifications of genetic polymorphism and phenotypic plasticity will be challenging tasks for the *A*. *auricular-judae* research and horticulture industries.

Six KEGG pathways were identified for significant relationships to the fruiting body wrinkle characteristics (Fig. 8, Supplementary Dataset S6). This result strongly suggested that wrinkle characteristics may not be controlled by a single gene. The presence of pathways of starch and sucrose metabolism, biosynthesis of amino acids and biosynthesis of secondary metabolites strongly supported the notion that food quality may vary among the cultivars with different wrinkle characteristics^6–8^. Biosynthesis of secondary metabolites is also important for the production and accumulation of pharmaceutical compounds, suggesting that different cultivars may have specific values of pharmaceutical development. Involvement of the biosynthesis of antibiotics pathway suggested difference in traits of disease resistance, therefore implicating potentials in breeding strategies.

In this study, an *A*. *auricular-judae* sequence library containing 13,937 unigenes was established and made publicly available (as a transcriptome shotgun assembly project that was deposited at DDBJ/EMBL/G under the accession GFZV00000000; see Supplementary Dataset S4 for the unannotated unigenes). Previously, only 13 *A*. *auricular-judae* gene sequences in our library had been available in public databases (Supplementary Table S3). Therefore, this work made a significantly knowledge advance for identifying *A*. *auricular-judae* gene expressions. The vast majority of these genes could be annotated with known GO functions, providing valuable information for understanding specific functions in future studies. Among the 1,124 unannotated unigenes that did not have significant sequencing similarities to known genes/proteins, expression of 627 unigenes was detected in all of the three varieties (Fig. 5b). These commonly expressed novel genes are likely to play roles in *A*. *auricular-judae* specific functions. In addition to its nutritional value in the food industry and novel enzymatic activities for the interests of biotechnology developments, *A*. *auricular-judae* fruiting bodies also produce bioactive molecules with pharmaceutical properties^1–3^, including anti-oxidant activities^32^. One of the particular encouraging areas is for anti-tumor activities^33–36^. Polysaccharides from *A*. *auricula-judae* inhibit the proliferation of Acinar cell carcinoma and induce apoptosis in S-180 tumor cells by upregulating the expression of *Bax* gene and down-regulating the Bcl-2 expression^37^. *A*. *auricula* polysaccharides also exhibit antiproliferative effects in HepG2 and Bel-7404 human hepatoma cells by inducing apoptosis and G1 or S cell cycle arrest^38^. Anti-proliferative activities, including high lactate dehydrogenase (LDH) levels, have been detected against human acute myelocytic leukemia U937 cells from *A*. *auricula-judae* extracts^39^. The novel genes, particularly those commonly expressed in all varieties (Fig. 4b), could be potential subjects for investigating anti-tumor activities.

To conclude, by analyzing the expression patterns of 13,937 *A*. *auricula-judae* unigenes, we determined the relationships among the three major cultivars in China. Supported by the expression patterns of all genes, annotated genes, un-annotated genes, differentially expressed genes and peroxidase genes, Quanjin displayed unique characteristics to Banjin and Wujin at the levels from specific gene function to global gene expression, indicating that the morphological difference of wrinkles in fruiting body associated with activities of many genes. Determination of altered activities in certain biological pathways associated with the wrinkle characteristics strongly suggested that fruiting bodies of the three cultivars contained different combinations of bioactive substances, supporting the implication that wrinkle characteristics may reflect nutritional and pharmaceutical properties.

## Methods

### Sampling, RNA extraction, and deep sequencing

Three varieties of *A*. *auricula-judae* known as Quanjin (Strain Hei29), Banjin (Strain Heibao), and Wujin (Strain Heishan) (Fig. 1) were collected from the “Huangsongdian National Station for *A*. *auricula-judae* Cultivation, Standardization and Demonstration”, Jiaohe City, Jilin Province, China, during the harvest season of autumn 2015. Individual fruiting bodies of each variety were sampled in triplicate, frozen in liquid nitrogen, and then stored at −80 °C prior to RNA extraction.

Total RNAs were isolated with TRIzol reagent (Invitrogen, USA) according to manufacturer’s protocols. The quality and quantity of RNA extracts were assessed using agarose gel electrophoresis and a Nanodrop 2000 Spectrophotometer (Thermo Scientific, USA). More than 5 µg of total RNA was obtained from each of the individual samples with OD_260/280_ > 1.8 and OD_260/230_ > 1.8.

The cDNA libraries were constructed for each individual sample using an Illumina TruSeq Stranded mRNA Library Preparation Kit LT for mRNA (Illumina, USA). The libraries were then individually sequenced using the manufacturer’s 125-nt pair-end protocol of the Illumina HiSeq platform at LC Sciences (China). Raw reads were filtered, and adapter sequences were removed by the manufacture’s quality control pipeline. The dataset has been deposited in NCBI/SRA database with accession numbers of SRX3295820 (Quanjin), SRX3295819 (Banjin), and SRX3304706 (Wujin).

### *De novo* assembly and unigene library

High quality reads were pooled for each of the varieties for *de novo* assembly using Trinity^23^. The resulting three contig libraries were clustered with TGICL-2.1^24^ by parameters of “-l 40 -v 20” to form a primary unigene library. It is worth noting that the unigene library does not represent polymorphisms although this method has been reported previously for comparative transcriptome analysis at intra-species level^40–47^.

All clean reads of each individual sample were mapped to the primary unigene library by bowtie-1.1.2^48^, respectively, with the following parameters “-q–phred33-quals -n 2 -e 99999999 -l 25 -I 1 -X 1000 -p 10 -a -m 200”. Then, the mapping output of each sample was calculated for read counts for each unigene by HTSeq-0.6.1p1^49^. The HTSeq outputs were further processed in edgeR^25,50^ to obtain the FPKM value for each unigene in each of the individual samples. For each variety, the median FPKM value of the three replicates was used to represent the expression level. To minimize possible experimental error, an expression threshold of FPKM > 1.0 was set to filter out weak expression. The unigenes with a median FPKM > 1.0 in any of the three varieties were used to form the final unigene library (Fig. 2).

### PCA

Raw read counts of the final unigene library were transformed to regularized log–transformed data by rlogTransformation^51^ to avoid domination of highly variable genes and to have a roughly equal contribution from all genes during the distance measuring. Gene expression cluster analysis was performed by heatmap.2. PCA was performed by the function prcomp of R. The principal component scores were plotted by qplot function of ggplot2 package.

### Annotation of unigenes

The NCBI-nt, NCBI-nr, and Swiss-prot databases were used to annotate the final *A*. *auricula-judae* unigene library. First, unigenes were searched against NCBI-nt by BLASTn, and the best hit (e-value < 1e-5) were retained as the nucleotide annotation. Second, the remaining unannotated unigenes were searched against the NCBI-nr database by BLASTx, and the best hits (e-value < 1e-4, identity > 30%) were selected as the protein annotation. Finally, the remaining unannotated unigenes were aligned to the Swiss-prot database using BLASTx with a cut-off threshold of e-value < 1e-4 and identity > 30%. The unigene sequences were deposited at DDBJ/EMBL/G under the accession number GFZV00000000.

### DEGs and GO functions

Based on the read counts, DEGs were identified using EdgeR (*P* < 0.05). Pairwise comparisons were performed among the three varieties. For each of the pairwise comparison, DEGs and unDEGs were separately recorded and used as inputs for GO annotations using Interproscan-5.2–45.0^52^ with the following parameter “ -t n -f tsv -goterms -pa -iprlookup -dp”. The GO annotations were collected. The number of unigenes for each GO term on Level-3 was calculated for the DEGs and unDEGs, respectively, by an in-house Perl program. The number of unigenes for a GO term was then normalized as Z-score ((x-mean)/(s.d.)) and represented in a heat map using an in-house Perl program.

### KEGG pathway enrichment associated with surface wrinkle

To identify genes that possibly associate with the fruiting body wrinkle characteristics, the transcriptome data (FPKM) for each unigene was ranked for the order of Wujin, Banjin and Quanjin, using the Short Time series Expression Miner (STEM) program^53^. Two sets of unigenes were extracted using FPKM values of Wujin > Banjin > Quanjin (wrinkle down-regulation) or Wujin < Banjin < Quanjin (wrinkle up-regulation). These two sets of unigenes were pooled then examined in KOBAS 3.0^54^ to identify gene enrichments in KEGG pathways, with default parameters and using *Auricularia delicata* as the reference species.

### Real time PCR validation of selected peroxidase-like unigene transcriptions

DNA primers of 16 selected peroxidase unigene targets and the *A*. *aj-DyP1* (NCBI Accession Number: JQ650250, Supplementary Figure S1) were designed using Primer Primer 5.0 software (http://www.premierbiosoft.com). Total RNA concentration was measured with Nanodrop 2000 (Thermo Fisher, USA) for each sample and 1 µg of RNA was used from each sample to synthesize the cDNA by the PrimeScript RT Master Mix kit (TaKaRa, Japan). Subsequently, quantitative (q)PCR was performed using the SYBR Premix Ex Taq kit (Tli RNaseH Plus) (TaKaRa, Japan) on a QuantStudioTM 7 Flex Real-Time PCR Detection System (Applied Biosystems, USA) following the manufacturer’s instructions. Each reaction was performed in a total volume of 20 µL with 1 µL of undiluted cDNA, 10 µL TB Green Premix Ex Taq II, 0.4 µL ROX Reference Dye II (SYBR Green, TaKaRa, Japan), 0.8 µL forward and reverse primer (10 µM), respectively. The PCR program was 95 °C for 10 min, 40 cycles (95 °C for 15 s, 60 °C for 30 s) with a single melting curve fluorescence measurement (60 °C–95 °C, +0.05 °C/s). Each experiment was performed in triplets with ddH2O replacing the cDNA as negative control. The Ct numbers were recorded and the qPCR results were analyzed using the ΔCt method using the 18 S rRNA as the reference gene^55^. Pearson correlation analysis was performed between ΔCt (median) and FKPM (median) values using MiniTab. All the RT-qPCR products of each cultivar were pooled for the agarose gel electrophoresis followed by gel excision and DNA purification using Gel Purification Kit (TIANGEN, China). The purified PCR products were sent for Sanger sequencing by GENEWIZ Corporation (SuZhou, China). The resulting DNA sequences were used to verify genuine amplifications of the target unigene fragments using local Blastn against the target unigene sequences.

## Supplementary information

Supplementary information

Dataset 1

Dataset 2

Dataset 3

Dataset 4

Dataset 5

Dataset 6

Dataset 7

Dataset 8

Dataset 9
