# Supplementary material for: Comparative transcriptome analysis reveals relationship of three major domesticated varieties of Auricularia auricula-judae
Source: Sci Rep. 2019 Jan 11;9:78. doi: 10.1038/s41598-018-36984-y (PMC6329756; doi:10.1038/s41598-018-36984-y)
Supplement: Supplementary file 1 — Supplementary information [file 41598_2018_36984_MOESM1_ESM.docx]

**Supplementary Material**

**Comparative transcriptome analysis reveals relationship of three major domesticated varieties of *Auricularia auricula-judae***

Yuhui Zhao^1,2#^, Liang Wang^1,2#^, Dongshan Zhang^3^, Rong Li^1^, Tianyou Cheng^4^, Yibi Zhang^4^, Xueju Liu^1^, Gary Wong ^5,6^，Yuguo Tang^1^, Hui Wang^1,7,8*^ & Shan Gao^1,4*^

1. CAS Key Laboratory of Biomedical & Diagnostic Technology, CAS/Suzhou Institute of Biomedical Engineering and Technology, Suzhou 215163, China

2. CAS Key Laboratory of Pathogenic Microbiology and Immunology, Institute of Microbiology, Chinese Academy of Sciences, Beijing 100101, China

3. Economic Cooperation Bureau, 11 Mingzhu Road, Jiaohe, Jilin 132500, China

4. Shanxi Academy of Advanced Research and Innovation, Taiyuan 030032, China

5. Shenzhen Key Laboratory of Pathogen and Immunity, Guangdong Key Laboratory for Diagnosis and Treatment of Emerging Infectious Diseases, Shenzhen Third People’s Hospital, Shenzhen 518112, China

6. Département de microbiologie-infectiologie et d’immunologie, Université Laval, Québec, QC, Canada

7. Institute of Biomedical Engineering, Old Road Campus, University of Oxford, Oxford OX3 7DQ, UK

8. Oxford Suzhou Centre for Advanced Research, 388 Ruoshui Road, Suzhou Industrial Park, Jiangsu 215123, China

#Yuhui Zhao and Liang Wang contributed equally to this work

*Correspondence to Hui Wang (huiwang789@gmail.com) and Shan Gao (gaos@sibet.ac.cn)

| **Supplementary Table S1: Summary of RNA-seq data** | | |
| --- | --- | --- |
| **Sample** | **Replicate** | **Clean data of transcriptome** |
|  |  |  |
| Banjin | 1 | 20,118,569 |
|  | 2 | 21,572,236 |
|  | 3 | 21,227,218 |
| Quanjin | 1 | 24,034,931 |
|  | 2 | 23,835,147 |
|  | 3 | 21,944,408 |
| Wujin | 1 | 23,217,478 |
|  | 2 | 25,682,704 |
|  | 3 | 20,689,481 |

**Supplementary Table S2:** **Summary of *Auricularia auricula-judae* transcriptome *de novo* assembly**

| Contig | Banjin | 29,523 |
| --- | --- | --- |
|  | Quanjin | 33,316 |
|  | Wujin | 33,682 |
|  | Total Number | 96,521 |
| Primary Unigenes | Total Number | 54,320 |
|  | Distinct Clusters | 20,129 |
|  | Distinct Singletons | 34,191 |
|  | Total Length | 85,318,513 |
|  | Mean Length | 1,571 |
|  | N50(nt) | 2,688 |
|  | Min Unigene Length(nt) | 201 |
|  | Max Unigene Length(nt) | 30,448 |
| Final unigenes | Total Number | 13,937 |
|  | Total Length | 36,153,923 |
|  | Mean Length | 2,594 |
|  | N50(nt) | 3,521 |
|  | Min Unigene Length(nt) | 201 |
|  | Max Unigene Length(nt) | 30,448 |

**Supplementary Table S3: Unigenes annotated to *A. auricula-judae* sequences in each of the nine samples**

|  |  | **Quanjin (FPKM)** | | | **Banjin (FPKM)** | | | **Wujin (FPKM)** | | |
| --- | --- | --- | --- | --- | --- | --- | --- | --- | --- | --- |
| **Unigene ID** | **Annotation Subject ID** | **Qj1** | **Qj2** | **Qj3** | **Bj1** | **Bj2** | **Bj3** | **Wj1** | **Wj2** | **Wj3** |
| Unigene1114 | gi\|440222357\|gb\|JQ929895.1 | 6.636 | 8.168 | 8.063 | 2.749 | 4.91 | 1.815 | 6.481 | 10.126 | 10.937 |
| Unigene995 | gi\|363987639\|gb\|JN712676.1 | 25600.2 | 23746.9 | 23937.5 | 50455.3 | 45881.1 | 50584.6 | 35952.8 | 29518.7 | 30766.3 |
| Unigene12819 | gi\|253740153\|gb\|GQ244321.1 | 6.348 | 2.825 | 2.131 | 0.528 | 2.08 | 0.343 | 0.24 | 0.568 | 0.741 |
| Unigene7969 | gi\|541129011\|gb\|KF135617.1 | 1.694 | 2.047 | 1.358 | 7.241 | 6.687 | 7.803 | 8.932 | 9.923 | 10.864 |
| Unigene10740 | gi\|541129011\|gb\|KF135617.1 | 1.181 | 1.145 | 2.306 | 0.235 | 0.267 | 0.396 | 0.358 | 0.384 | 0.643 |
| Unigene6711 | gi\|284813609\|gb\|GU370887.1 | 41.219 | 43.268 | 45.325 | 35.262 | 30.849 | 50.622 | 34.348 | 18.133 | 42.18 |
| Unigene10769 | gi\|328684524\|gb\|JF440751.1 | 7.88 | 8.913 | 10.891 | 0.336 | 0.219 | 0 | 0.023 | 0.021 | 0 |
| Unigene6013 | gi\|40218019\|gb\|AY485828.1 | 74.967 | 70.643 | 70.999 | 59.328 | 101.912 | 71.037 | 48.435 | 42.402 | 62.688 |
| Unigene8681 | gi\|346995725\|gb\|JN628865.1 | 36.965 | 33.229 | 34.487 | 115.002 | 145.541 | 108.665 | 198.051 | 279.411 | 321.801 |
| Unigene5106 | gi\|440222356\|gb\|JQ929894.1 | 204.83 | 211.361 | 237.016 | 44.562 | 56.49 | 40.678 | 83.029 | 121.763 | 153.786 |
| Unigene1411 | gi\|924859\|gb\|U27022.1\|AAU27022 | 2.877 | 2.343 | 2.915 | 1.68 | 4.048 | 3.125 | 9.719 | 12.86 | 8.52 |
| Unigene10930 | gi\|633865329\|gb\|KC991156.1 | 2.634 | 4.568 | 4.16 | 0.818 | 0.154 | 1.698 | 0.594 | 1.072 | 0.501 |
| Unigene2997 | gi\|328684530\|gb\|JF440757.1 | 6.388 | 8.991 | 6.35 | 4.953 | 9.311 | 11.872 | 6.184 | 5.649 | 8.365 |
| Percentage of all unigenes (%) | | 0.0986 | 0.0988 | 0.0989 | 0.101 | 0.101 | 0.0935 | 0.101 | 0.101 | 0.0932 |

**Supplementary Table S4: Number of differentially expressed unigenes determined by pairwise comparisons**

| **DEG (up-\down-regulation)** | **Quanjin** | **Banjin** | **Wujin** |
| --- | --- | --- | --- |
| Quanjin (up-regulation) | - | 3231 | 3493 |
| Banjin (up-regulation) | 2761 | - | 1405 |
| Wujin (up-regulation) | 3056 | 1456 | - |

**Supplementary Table S5: RT-qPCR validation of expression levels of selected peroxidase-like unigenes.** The median of three replicates was represented.

|  | Quanjin | | Banjin | | Wujin | |
| --- | --- | --- | --- | --- | --- | --- |
| Unigene ID | FPKM | △Ct | FPKM | △Ct | FPKM | △Ct |
| 7660 | 124.7998 | 15.0948 | 94.5200 | 14.4459 | 127.1621 | 15.3585 |
| 7271 | 0.4280 | 22.7765 | 10.0487 | 16.4673 | 2.9112 | 16.6717 |
| 11260 | 1.5633 | 20.1877 | 0.4509 | 21.0510 | 2.4867 | 21.8181 |
| 13933 | 0.6681 | 19.7159 | 0.6268 | 20.4395 | 1.1642 | 21.2939 |
| 4425 | 87.6534 | 14.6965 | 47.1127 | 16.4519 | 69.4897 | 16.7656 |
| 13643 | 0.0937 | 20.5338 | 0.8330 | 20.8187 | 1.5342 | 21.2608 |
| 6898 | 1.5245 | 18.6054 | 1.0019 | 19.3896 | 0.6699 | 19.1280 |
| 2620 | 1.6307 | 17.7507 | 1.6375 | 17.6469 | 0.9189 | 18.7860 |
| 8988 | 3.6560 | 19.6434 | 3.1096 | 19.3661 | 4.1211 | 20.8066 |
| 10955 | 1.1594 | 21.2045 | 0.0510 | 22.4718 | 0.2654 | 23.7817 |
| 10269 | 5.5726 | 20.6630 | 0.0177 | 22.8817 | 0.0000 | 22.7393 |
| 3662 | 7.0862 | 24.7228 | 5.7940 | 24.6724 | 5.1228 | 24.4028 |
| 2337 | 0.0229 | 26.0299 | 6.4121 | 20.5763 | 7.4733 | 20.1450 |
| 11367 | 1.0968 | 18.7271 | 0.1802 | 20.8453 | 0.2086 | 19.3608 |
| 12290 | 1.0713 | 28.7565 | 0.0000 | Undetected | 0.0836 | Undetected |
| 11083 | 1.6153 | 17.8463 | 0.0000 | Undetected | 0.0918 | Undetected |
| *R* | -0.5641 | | -0.6947 | | -0.6451 | |
| *P* | 0.023 | | 0.006 | | 0.013 | |

**Supplementary Table S6: Expression of *A.aj_DyP1***

| **Local Blastn for A.aj_DyP1 (JQ650250)** | | | | | | | | | | | |
| --- | --- | --- | --- | --- | --- | --- | --- | --- | --- | --- | --- |
| **Query ids** | **Subject ids** | **Identity** | **Alignment length** | **Misma**  **tches** | **Gap opens** | **Q. start** | **Q.end** | **S. start** | **S. end** | **Evalue** | **Bit score** |
| CL10230Contig1 | JQ650250.1 | 99.75 | 803 | 2 | 0 | 1 | 803 | 24 | 826 | 0 | 1576 |
| CL15062Contig1 | JQ650250.1 | 99.31 | 722 | 5 | 0 | 1 | 722 | 962 | 1683 | 0 | 1392 |

| **Expression levels (Median)** | | | |
| --- | --- | --- | --- |
|  | **FPKM** | | |
| **Gene ID** | **Quanjin** | **Banjin** | **Wujin** |
| CL10230  Contig1 | 0.228656 | 0 | 0.14138 |
| CL15062  Contig1 | 0.156325 | 0.086821 | 0 |
| **ΔCt** | | | |
| JQ650250 | 24.8114 | 25.82571 | 23.40767 |

**Supplementary Figure S1: Phylogenetic tree of peroxidase-related unigenes**





Maximum likelihood trees were constructed using alignments of *A. auricula-judae* and *Auricularia subglabra*, nucleotide sequences for HTP peroxidase (Panel a), DyP-type peroxidase (Panel b), and MnP-atypical short manganese peroxidase (Panel c) using the *Leptoxyphium fumago* HTP1 (KX289328), *E. glandulosa* DyP1 (JQ650251), and MnP (JQ654420) sequences as the root, respectively. Bootstrap values ( > 70% ) are labeled on the tree. Filled diamonds labelled *A. auricula-judae* unigenes included in the final unigene set and an open diamond labelled the *A.aj-DyP1* transcription detected in this study but not included in the final unigene set due to the filtration of FPKM > 1.0.
