# Supplementary material for: Comparative transcriptome analysis reveals relationship of three major domesticated varieties of Auricularia auricula-judae
Source: Sci Rep. 2019 Jan 11;9:78. doi: 10.1038/s41598-018-36984-y (PMC6329756; doi:10.1038/s41598-018-36984-y)
Supplement: Supplementary file 9 — Dataset 8 [file 41598_2018_36984_MOESM9_ESM.docx]

Dataset S8: Sanger sequences of selected peroxidase-like unigene fragments

>Unigene7660_Banjin

CTNTNTCCNNNTACCTCAGGCGGGCGATGCGAGAGGTCCCTGCCCCGGCATGAACACACTCGCAAACCACGGCTGGTTGCCTCGTTCGGGAGTCGCCACCCCACAGCAGATCATCACCGCCTCGGAGAAAGGCTTCAACATGGGTCACAACCTAGCTACATTCGTCACTTTCGCGAACATGCTTGTGAACGGAAACCATCTGACGAACCTGATGAGCATCGGCACTCTCACCCCCCTCACGGGCCCAGAGCCGCCGAAGCCCGCCATCGCGGGTGGTCTCAGCACTCACAACACCTTCGAAGGTGATGCGAGCTGGACGAGACTAA

>Unigene7660_Quanjin

GNNNNANGGCTNNCTCAGACGGGCGATGCGAGAGGTCCACTGCCCCGGCATGAACACACTCGCAAACCACGGCTGGTTGCCTCGTTCGGGAGTCGCCACCCCACAGCAGATCATCACCGCCTCGGAGAAAGGCTTCAACATGGGTCACAACCTAGCTACATTCGTCACTTTCGCGAACATGCTTGTGAACGGAAACCATCTGACGAACCTGATGAGCATCGGCACTCTCACCCCCCTCACGGGCCCAGAGCCGCCGAAGCCCGCCATCGCGGGTGGTCTCAGCACTCACAACACCTTCGAAGGTGATGCGAGCATGACGAGACTAA

>Unigene7660_Wujin

CTTNNCCNNNGCTCAGGCGGGCGATGCGAGAGGTCCCTGCCCCGGCATGAACACACTCGCAAACCACGGCTGGTTGCCTCGTTCGGGAGTCGCCACCCCACAGCAGATCATCACCGCCTCGGAGAAAGGCTTCAACATGGGTCACAACCTAGCTACATTCGTCACTTTCGCGAACATGCTTGTGAACGGAAACCATCTGACGAACCTGATGAGCATCGGCACTCTCACCCCCCTCACTGGCCCAGAGCCGCCGAAGCCCGCCATCGCGGGTGGTCTCAGCACTCACAACACCTTCGAAGGTGATGCGAGCTGACGAGACTAA

>Unigene7271_Quanjin

NNNGNNTNNCAGGNACTCGCNCNNTCATGCACGAGGCTAGCAAGCAGTTCGGTGGCGGCAAATACAACTTGAGCGCCGCTGCTGAACACCGCTGGAACCGTATCCAAGACTCTATCGCACGCAACCCGACCTTCCAGTTCAGCAACCCGCGCTTTGCCACAGCGTATGTTGAGGCCGTCTTCCCGATAGCCTTCTTCATCGACGGCCATGTCAAAGGCGACGACGTGCGCGGTGCGGAGGGACTGGACCTTGACATCATGCGCGGCTTCTTCCAGGACTCGCGCAAAGGGACTGGACCTTGACATCATGCGCGGCTTCTTCCAGGACTCGCGCAA

>Unigene7271_Banjin

NNNGNCNTNNNAGACGCTCTTTGACCAGATCGTCGAGGCTAGCAAGCAGTTCGGTGAGCGGCAAATACAACTTGAGCGCCGCTGCTGAACACCGCTGGAACCGTATCCAAGACTCTATCGCACGCAACCCGACCTTCCAGTTCAGCAACCCGCGCTTTGCCACAGCGTATGTTGAGGCCGTCTTCCCGATAGCGTTCTTCATCGACGGCCATGTCAAAGGCGACGACGTGCGCGGTGCGGAGGGACTGGACCTTGACATCATGCGCGGCTTCTTCCAGGACTCGCGCAA

>Unigene7271_Wujin

NNNGNNTANTAGACGCTCTTTGACCAGATCGTCGAGGCTAGCAAGCAGTTCGGTGGCGGCAAATACAACTTGAGCGCCGCTGCTGAACACCGCTGGAACCGTATCCAAGACTCTATCGCACGCAACCCGACCTTCCAGTTCAGCAACCCGCGCTTTGCCACAGCGTATGTTGAGGCCGTCTTCCCGATAGCGTTCTTCATCGACGGCCATGTCAAAGGCGACGACGTGCGCGGTGCGGAGGGACTGGACCTTGACATCATGCGCGGCTTCTTCCAGGACTCGCGCAA

>Unigene11367_Wujin

NNNNNNNNNNNCNGCGCGATGGTACTCCTGGATACGGCGCCAGCGGAGTTCGCCCGCGACGGTCAGGTTGTACCTCCCAGCACCGAACTTCTTGCTGTCTTTCTCGATCAGCTTGAACAGCGTGTTGTTGAAGCTGTGGTTATCGCCCAAGAAGTCATCGCTGCGCGTGAGACTCGCGTCGCCTTCGAAAGTGCCGTGAACGTTGAGACCGCCAGCGAGCGCTGGCTTCGGGGGCGCAAGCCCGGTCAACGGCGAGAGACCACCGATGCTCATCAAGTTGGTGA

>Unigene11367_Quanjin

CAANANNTANNNNNTGCGCGATGGACTCCTGGATACGGCGCCAGCGGACTTCGCCCGCGACGGTCAGGTTGTACCTCCCAGCACCGAACTTCTTGCTGTCTTTCTCGATCAGCTTGAACAGCGTGTTGTTGAAGCTGTGGTTATCGCCCAAGAAGTCATCGCTGCGCGTGAGACTCGCATCGCCTTCGAAAGTGCCGTGAACGTTGAGACCGCCAGCGAGCGCTGGCTTCGGGGGCGCAAGCCCGGTCAACGGCGAGAGACCACCGATGCTCATCAAGTTGGTGA

>Unigene11367_Banjin

NNGNNNCCCTNTGNGCGTATGGTCTCCTGGATGCGGCGCCGCGGACTTCGCCCGCGAGCGGTCAGGTTGTACCTCCCAGCACCGAACTTCTTGCTGTCTTTCTCGATCAGCTTGAACAGCGTGTTGTTGAAGCTGTGGTTATCGCCCAAGAAGTCATCGCTGCGCGTGAGACTCGCGTCGCCTTCGAAAGTGCCGTGAACGTTGAGACCGCCAGCGAGCGCTGGCTTCGGGGGCGCAAGCCCGGTCAACGGCGAGAGACCACCGATGCTCATCAAGTTGGTGA

>Unigene11260_Quanjin

NNNNAANTCGCGCTCGCGATGCGACTTCTACTTGTGCGCAGGGGACAATCACAACATGCAGCCCGATCTCTTCCGGCAGATGCTGGAGCGCGCTGAGGCGCATGGTAACCAGTTCAACGTAGCCGCGCTGCAGGAACACTTCCACAATCGTTACGCACACTCCAGGGAGAACAACCCCAACTTCTACTTTGTTCCCCCGTCTGGTGTTGTTACCATGGGCGCAACTTATTTCCACGCCGGTTTCTTCACCAACGGCACAATTGGCTACGGAGGCTCCTCCAACGTCGCGTCCATCTCTAAA

>Unigene11260_Banjin

NNNGNNACGCGCTCCGATGCGACTTCTACTTGTGCGCAGGGGACAATCACAACATGCAGCCCGATCTCTTCCGGCAGATGCTGGAGCGCGCTGAGGCGCATGGTAACCAGTTCAACGTAGCCGCGCTGCAGGAACACTTCCACAATCGTTACGCACACTCCAGGGAGAACAACCCCAACTTCTACTTTGTTCCCCCGTCTGGTGTTGTTACCATGGGCGCAACTTATTTCCACGCCGGTTTTTTCACCAACGGCACAATTGGCTACGGAGGCTCCTCCAACGTCGCGTCCATCTCTTTAACGACGGCGATTTGGCTCGGAGGCCCCCACGCCGCCTCTCTAAN

>Unigene11260_wujin

NNNNNNNCGTNGTNNCCGATGCGACTTCTACTTGTGCGCAGGGGACAATCACAACATGCAGCCCGATCTCTTCCGGCAGATGCTGGAGCGCGCTGAGGCGCATGGTAACCAGTTCAACGTAGCCGCGCTGCAGGAACACTTCCACAATCGTTACGCACACTCCAGGGAGAACAACCCCAACTTCTACTTTGTTCCCCCGTCTGGTGTTGTTACCATGGGCGCAACTTATTTCCACGCCGGTTTTTTCACCAACGGCACAATTGGCTACGGAGGCTCCTCCAACGTCGCGTCCATCTCTTAA

>Unigene2337_Banjin

NNNNCNNANNTTATTTAGGAGTCGTATAGTAGTATACAATTAGCTGCAGTGTGAGTAGATTATTGTACATTCCCACGAATCCATGTCATTAGTCGTCATTCAGTCTGATCTGTCGTGGTCCCCGCTGCTCTACGATTCCCAGCTCGACGAATACGGCGGTCACATTGATATTGAATATGGTGATGCCGAACCGGGAGCGATTGCTAGGCTCCCAACCCTCAGGCAGGCGCTCTTCTCGGAGGATGGGCTCCAGGTCGCGCACATTGA

>Unigene2337_Quanjin

NNNGNNNCNNATTTAGGAGTCGTATAGTAGTATACAATTACATGCAGTGTGAGTAGATTATTGTACATTCCCACAAATCCATGTCATTAGTCGTCATTCAGCCTGATCTGTCGTGGTCCCCGCTGCTCTACGATTCCCAGCTCGACGAATACGGCGGTCACATTGATGTTGAATATGGTGATGCCGAACCGGGAGCGATTGCTAGGCTCCCAACCCTCAGGCAGGCGCTCTTCTCGGAGGATGGGCTCCAGGTCGCGCACATTGA

>Unigene2337_Wujin

TNNGNNNGNTATNTTAGGAGTCGTATAGTAGTATACAATTAGCTGCAGTGTGAGTAGATTATTGTACATTCCCACGAATCCATGTCATTAGTCGTCATTCAGTCTGATCTGTCGTGGTCCCCGCTGCTCTACGATTCCCAGCTCGACGAATACGGCGGTCACATTGATATTGAATATGGTGATGCCGAACCGGGAGCGATTGCTAGGCTCCCAACCCTCAGGCAGGCGCTCTTCTCGGAGGATGGGCTCCAGGTCGCGCACATTGA

>Unigene4425_Banjin

NNNNNNNCAGCGCTCGTNGGCGATCTACGGACTGTCGCGCTTTTTCGCCGTCGTTCTTGCCTACGGCGCCTTCTTAGGCGTGAACCTGGGCAACAAGCACCGGTTTTCGTTCACACTGGACATGAGCGACCTCTGCTCCAAGGGCGTCCCGCATCCAGCGCCTTTAGTACACGAAAATCCGCATCCAGTCACGCTCTCCGTCAAAGGACGGCCCAGCGCGTACCTTACGAACAAAGTGATCGAGCTCGCGGAGGACGCGCCAGACGTCAATCTCCAACACTTCGCGCACTGGCGGATAAA

>Unigene4425_Quanjin

GNGANAANGNCAANNCTCGTTGGCGTCTACGGACTGTCGCGCTTTTTCGCCGTCGTTCTTGCCTACGGCGCCTTCTTAGGCGTGAACCTGGGCAACAAGCACCGGTTTTCGTTCACACTGGACATGAGCGACCTCTGCTCCAAGGGCGTCCCGCATCCAGCGCCTTTAGTACACGAAAATCCGCATCCAGTCACGCTCTCCGTCAAAGGACGGCCCAGCGCGTACCTTACGAACAAAGTGATCGAGCTCGCGGAGGACGCGCCAGACGTCAATCTCCAACACTTCGCGCACTGGCGGATAAA

>Unigene4425_Wujin

GGNNNNGNNCANNGCTCGTTGGCGTCTACGGACTGTCGCGCTTTTTCGCCGTCGTTCTTGCCTACGGCGCCTTCTTAGGCGTGAACCTGGGCAACAAGCACCGGTTTTCGTTCACACTGGACATGAGCGACCTCTGCTCCAAGGGCGTCCCGCATCCAGCGCCTTTAGTACACGAAAATCCGCATCCAGTCACGCTCTCCGTCAAAGGACGGCCCAGCGCGTACCTTACGAACAAAGTGATCGAGCTCGCGGAGGACGCGCCAGACGTCAATCTCCAACACTTCGCGCACTGGCGGATAAA

>Unigene13643_Banjin

NNNANCTGTNCGACATTTCGTGACCAGCCAGGTCTCCGGGGCGCATCGAAGCATCGAGTGAGTACACCTTGCTGAGCGACGAGCCGAATGTGCTCTCAATGAAGGCGACTTGCTGGTTGATGAGATCGGAGGTGTCGCTCGCGAGGAGCATCACGCCGTGGATACCAGTCCCTTTGAACTCCTGCTGCATCCGTCCGACGTCTCCGCGAGCGTCTGGATGGCCTGGAGCTGGCCGAGGGTGAAGGATGCATCGCCAACGTCGTCAAGGACCCCCAGCGCGGTGAGGCCAGACTGGGAGAACGCAACA

>Unigene13643_Quanjin

NNNNCNNGNNCGACTTTCGTGACCAGCCAGGTCTCCGGGGCGCATCGAAGCATCGAGTGAGTACACCTTGCTGAGCGACGAGCCGAAGGTGCTCTCAATGAAGGCGACTTGCTGGTTTATGAGATCGGAGGTGTCGCTCGCGAGGAGCATCACGCCGTGGATACCAGTCCCTTTGAACTCCTGCTGCCATCCGTCCGACGTCTCCGCGAGCGTCTGGATGGCCTGGAGCTGGCCGAGGGTGAAGGATGCATCGCCGACGTCGTCAAGGACCCCCAGCGCGGTGAGGCCAGACTGGGAGAACGCAACCACCGACGTCGTCAAGGACCCCCAGCGCGGTGAGGCCAGACTGGGAGAACGCAACA

>Unigene13643_Wujin

NNNANCANGNTGACGACATTTCGTGACCAGCCAGGTCTCCGGGGCGCATCGAAGCATCGAGTGAGTACACCTTGCTGAGCGACGAGCCGAATGTGCTCTCAATGAAGGCGACTTGCTGGTTGATGAGATCGGAGGTGTCGCTCGCGAGGAGCATCACGCCGTGGATACCAGTCCCTTTGAACTCCTGCTGCCATCCGTCCGACGTCTCCGCGAGCGTCTGGATGGCCTGGAGCTGGCCGAGGGTGAAGGATGCATCGCCAACGTCGTCAAGGACCCCCAGCGCGGTGAGGCCAGACTGGGAGAACGCAACAA

>Unigene10955_Banjin

NNNNNNNNCNNGNANCNNAGACNTGNAGNTNAGATTGTTCTACTTCTTCGCCATATCAGGATGTGGCCACGTTCAAGACGCATCTCGCGAACGACATCGCGCCAGTCCTCACCTCGGCCGAGCAGATGATGAACATGACGCAGCAACCCCTCGTCCTCTTGAACGTTGCGTTCTCCCAGTCTGGCCTCACCGCGCTGGGGGTCCTTGACGACGTTGGCGATGCATCCTTCACCCTCGGCCAGCTCCAGGCCATCCAGACGCTCGCGGAGACGTCGGACGGATGGCAGCAGGAGTTCAAAGGGACTGGTATCCACGGCGTGATGCTCCTCGCGAGCGACACCTCCGATCTCATAA

>Unigene10955_Quanjin

TNNAANNNAGTCNNATNANCNTATAGTNTAANATTGTTCTACTTCTTCGCCATTCAGGATGTGGCCACGTTCAAGACGCATCTCGCGAACGACATCGCGCCAGTCCTCACCTCGGCCGAGCAGATGATGAACATGACGCAGCAACCCCTCGTCCTCTTGAACGTCGCGTTCTCCCAGTCTGGCCTCACCGCGCTGGGGGTCCTTGACGACGTCGGCGATGCATCCTTCACCCTCGGCCAGCTCCAGGCCATCCAGACGCTCGCGGAGACGTCGGACGGATGGCAGCAGGAGTTCAAAGGGACTGGTATCCACGGCGTGATGCTCCTCGCGAGCGACACCTCCGATCTCATAA

>Unigene10955_Wujin

TAGCTNNNNAGACGTGCAGNTNNNGATTGTTCTACTTCTTCGCCATTCAGGATGTGGCCACGTTCAAGACGCATCTCGCGAACGACATCGCGCCAGTCCTCACCTCGGCCGAGCAGATGATGAACATGACGCAGCAACCCCTCGTCCTCTTGAACGTTGCGTTCTCCCAGTCTGGCCTCACCGCGCTGGGGGTCCTTGACGACGTTGGCGATGCATCCTTCACCCTCGGCCAGCTCCAGGCCATCCAGACGCTCGCGGAGACGTCGGACGGATGGCAGCAGGAGTTCAAAGGGACTGGTATCCACGGCGTGATGCTCCTCGCGAGCGACACCTCCGATCTCATAA

>Unigene2620_Banjin

NNCNNGNNNAGTGNANTGTGCACTCGCATGCCCGGCAAAGAGGGTTAAAACGATGACGAGCATACCCGCTATGAGGCTCGCGGCGAAGAGGCCCAGGATAGTGATGACTGCCCTACTATCGCGCTCGTAGAGTGCACATGTCCTGTAGCATAACACCGCTCCTGGAATAGCAAACCACTCGATTATTGCTGCAGTGGACATCACATCCTTGATCATCGCTGTACAGAATTCGTCGGACACCTTGGGATATGTCAGACCAATTACGTTGAAGAGCTGGTGAGCATCA

>Unigene2620_Quanjin

TNNNNNCGGTGNNCTGTGCACTCGCATGCCCGGCAAGAGGGTTAAAACGATGACGAGCATACCCGCTATGAGGCTCGCGGCGAAGAGGCCCAGGATAGTGATGACTGCCCTACTATCGCGCTCGTAGAGTGCACATGTCCTGTAGCATAACACCGCTCCTGGAATAGCAAACCACTCGATTATTGCTGCAGTGGACATCACATCCTTGATCATCGCTGTACAGAATTCGTCGGACACCTTGGGATATGTCAGACCAATTACGTTGAAGAGCTGGTGAGCATCA

>Unigene2620_Wujin

NNNNNNNNNNGNGNANTGTGCACTCGCATGCCCGGCAAGAGGGTTAAAACGATGACGAGCATACCCGCTATGAGGCTCGCGGCGAAGAGGCCCAGGATAGTGATGACTGCCCTACTATCGCGCTCGTAGAGTGCACATGTCCTGTAGCATAACACCGCTCCTGGAATAGCAAACCACTCGATTATTGCTGCAGTGGACATCACATCCTTGATCATCGCTGTACAGAATTCGTCGGACACCTTGGGATATGTCAGACCAATTACGTTGAAGAGCTTGTGAGCATCA

>Unigene8988_Banjin

NTNACNANCGCAGCACGTTGAGCGAGCGTCTTACCTTCAAAGGGCGGCGCGTTGTCGAGCATGAACTTGTTGAATTCAGGCACGAGCTGCTGCAGCTGACGGAAGACAAGGAAGGAACCGCCGGTTGCCCAGGCGGGCCGTGCGATTGCGTCATTGGTTGCACCTACGATGATAACACCAGGATCGACCACAATCTGACCCGGCAATGGATCATGGAACCCATTCAGAGCTGGTTGCGCGATACCGTCGACAAAGCCGAAGGGTTCGTGACCAGCCTCGGCGCCTGGACGGACAGAGGCGCGAGAGTATGAA

>Unigene8988_Quanjin

NNGANNNNNANAGNACGCTGCANCGAGCGTCTTACCTTCAAAGGGCAGGCGCGTTGTCGAGCATGAACTTGTTGAATTCAGGCACGAGCTGCTGCAGCTGACGGAAGACAAGGAAGGAACCGCCGGTTGCCCAGGCGGGCCGTGCGATTGCGTCATTGGTTGCACCTACGATGATAACACCAGGATCGACCACAATCTGACCCGGCAATGGATCATGGAACCCATTCAGAGCTGGTTGCGCGATACCGTCGACAAAGCCGAAGGGTTCGTGACCAGCCTCGGCGCCTGGACGGACAGAGGCGTCGAGAGTATGA

>Unigene8988_Wujin

NNNNTCNANNNGCGCACTNTAGAGTANAGACGTCTTACCTTCAAAGGGCGGCGCGTTGTCGAGCATGAACTTGTTGAATTCAGGCACGAGCTGCTGCAGCTGACGGAAGACAAGGAAGGAACCGCCGGTTGCCCAGGCGGGCCGTGCGATTGCGTCATTGGTTGCACCTACGATGATAACACCAGGATCGACCACAATCTGACCCGGCAATGGATCATGGAACCCATTCAGAGCTGGTTGCGCGATACCGTCGACAAAGCCGAAGGGTTCGTGACCAGCCTCGGCGCCTGGACGGACAGAGGCGTCGAGAGTATGA

>Unigene6898_Banjin

NNNTANAANGAGTCAGCATACTGGAAAGAGCGGATGCACAAGGCGAATTAGGCCGGGACGGCGACTCATCGAGACTGTCAGAGTCAAGCTTAAACTCGTTGTAATTGATTAAAAGGAGCTTTCAGTGGCGACACTGCGGCGACAATGGGCGTGTGATTATGAACGGTCCCCGGATAGCTCGCAGTTCCTTGTCGTCGGTGGCAATTATTCTACGGCCATATAGCAGTTGACAGTGTTCAGGAAGTTCAGCAATGCCTTTCGCTGCGTCCAGGAAAATCGTGAAATTGGGAGGCTCCAGTTAAAAACCTGTGCTAGCGCGCGAGACGCGAGGATTGGGTCA

>Unigene6898_Quanjin

NNNGTAGNANATGAGTCATGCATACTGGAAAGAGCGGATGCACAAGGCGAATTAGGCCGGGACGGCGACTCATCGAGACTGTCAGAGTCAAGCTTAAACTCGTTGTAATTGATTAAAAGGAGCTTTCAGTGGCGACACTGCGGCGACAATGGGCGTGTGATTATGAACGGTCCCCGGATAGCTCGCAGTTCCTTGTCGTCGGTGGCAATTATTCTACGGCCATATAGCAGTTGACAGTGTTCAGGAAGTTCAGCAATGCCTTTCGCTGCGTCCAGGAGAATCGTGAAATTGGGAGGCTCCAGTTAAAAACCTGTGCTAGCGCGCGAGACGCGAGGATTGGGTCA

>Unigene6898_Wujin

NNGCCNNNNATGAGTCAGAGCATACTGGATCAGAGCGGATCGCACAAGCGTCGAATCTACGGCCAGGGACGGCTACTCATCAGAGACTGTCAGAGTCAAGCTTAAACTCGTTGTAATTGATTAAAAGGAGCTTTCAGTGGCGACACTGCGGCGACAATGGGCGTGTGATTATGAACGGTCCCCGGATAGCTCGCAGTTCCTTGTCGTCGGTGGCAATTATTCTACGGCCATATAGCAGTTGACAGTGTTCAGGAAGTTCAGCAATGCCTTTCGCTGCGTCCAGGAGAATCGTGAAATTGGGAGGCTCCAGTTAAAAACCTGTGCTAGCGCGCGAGACGCGAGGATTGGGTCAA

>Unigene11083_Quanjin

NNNCNGNCAATCATGACGACNTCGAGCAGGGCTGCGCGACGCAAGCCTTCCCGACGACGCTCTCCACCGCCGCTGGGCAGCCCACTCCCATCCCGCCTGTCAACGTGCAGGATATCGCGTTTGGCAGGAAGTGATTGATCTCCATCTTCAGTATCTACAGTACTAGATGTGTCGTGGCTTTCTCGAATTGGTTTTTGTTATTGCTTTTTCAACTTTGTCGTCCTCAATTTTGTTTTTGGTACTCGCAGTGCGATTCATTTGTTTCCTATTCCATTCTCAACTCACGA

>JQ650250_Quanjin

NNNNNNTCNCACNGNCAGGCCAAGATGCCACCAGCTTCAAGGAATCAACCTCGTCATGGGTCCCTCAGTTTGCCGGCACTGGGATCCACGGCGTCATCATCCTCGCGAGCGACACGACCGACCTCATTGACCAGCAGGTGGCTTCGATTGAGTCGACTTTCGGCTCGTCGATCTCGAAGCTCTACTCGTTGTCGGCTTCGATCCGTCCAGGAAACGAAGCTGGTCATGAAATGTTCGGCTTCCTTGATGGAATTGCTCAGCCCGCCATCAACGGCTTTAACACTCCTCTGCCTGAG

>JQ650250_Banjin

NNNNNNGCACGGNNGGCCATAGATGCCACCAGCTTCAAGGAATCAACCTCGTCATGGGTCCCTCAGTTTGCCGGCACTGGGATCCACGGCGTCATCATCCTCGCGAGCGACACGACCGACCTCATTGACCAGCAGGTGGCTTCGATTGAGTCGACTTTCGGCTCGTCGATCTCGAAGCTCTACTCGTTGTCGGCTTCGATCCGTCCAGGAAACGAAGCTGGTCATGAAATGTTCGGCTTCCTTGATGGAATTGCTCAGCCCGCCATCAACGGCTTTAACACTCCTCTGCCTGA

>JQ650250_Wujin

NNANNNTTTGCACGGNAGGCCAAGATGCCACCAGCTTCAAGGAATCAACCTCGTCATGGGTCCCTCAGTTTGCCGGCACTGGGATCCACGGCGTCATCATCCTCGCGAGCGACACGACCGACCTCATTGACCAGCAGGTGGCTTCGATTGAGTCGACTTTCGGCTCGTCGATCTCGAAGCTCTACTCGTTGTCGGCTTCGATCCGTCCAGGAAACGAAGCTGGTCATGAAATGTTCGGCTTCCTTGATGGAATTGCTCAGCCCGCCATCAACGGCTTTAACACTCCTCTGCCTGA

>Unigene10269_Banjin

NNNNCACTCGCGCAGACGCCTTCGAGAGGTCGTTGATGAAGTCGTCGCGGATGGCAGCCCATGCGCAGCACTGAACATCCTTCACGCGCTCCTTGCTGTTGGGGCAAGTGTGAAGGGCATCCGGCAACGTGGCACCAAACGCAAGGCGTGCTGCCGCCAGAGTCACAACGAACGCGCGGAGAACCGTGAAGGACATGGTCGAGGACGAGTCGAGGGAGGGAGCTGAGAGAACGAGAAACGAATCGCAGGACTCAACTTTATACTCGAGACCCCTGCTTTCCCGGGTATGTGAGATCTCGACAACAAGCGCAGATTATTGAC

>Unigene10269_Quanjin

NNNGACAGCNTCGCCGCAGACGCCTTCGAGAGGTCGTTGATGAAGTCGTCGCGGATGGCAGCCCATGCGCAGCACTGAACATCCTTCACGCGCTCCTTGCTGTTGGGGCAAGTGTGAAGGGCATCCGGCAACGTGGCACCAAACGCAAGGCGTGCTGCCGCCAGAGTCACAACGAACGCGCGGAGAACCGTGAAGGACATGGTCGAGGACGAGTCGAGGGAGGGAGCTGAGAGAACGAGAAACGAATCGCAGGACTCAACTTTATACTCGAGACCCCTGCTTTCCCGGGTATGTGAGATCTCGACAACAAGCGCAGATTATTGACA

>Unigene10269_Wujin

NNNNNCTTCGCGCAGACGCCTTCGAGAGGTCGTTGATGAAGTCGTCGCGGATGGCAGCCCATGCGCAGCACTGAACATCCTTCACGCGCTCCTTGCTGTTGGGGCAAGTGTGAAGGGCATCCGGCAACGTGGCACCAAACGCAAGGCGTGCTGCCGCCAGAGTCACAACGAACGCGCGGAGAACCGTGAAGGACATGGTCGAGGACGAGTCGAGGGAGGGAGCTGAGAGAACGAGAAACGAATCGCAGGACTCAACTTTATACTCGAGACCCCTGCTTTCCCGGGTATGTGAGATCTCGACAACAAGCGCAGATTATTGACA

>Unigene13933_Banjin

ANANTNNNTNAGATCTTCTTGGTCTCTGTCAATTTCTTAAGCTCTCCGAGCTGTCAGCACGATACTGTCTGCTAACGTGATAGAGAGATGGCCGTCTCTACAACGACGACAAAATGGTCTCAAACTGTTGTCGGGGGATTTCTCCTCGGAAACGCTGGTGGTCAGTTGGAATGACCGTTAACCGCTTTGAACTGACACTGGCAGAGCTGATTGAAATACATATGCAGCCGCCTAAGGAGCGAAGGCCATACACATGTAGATATAGTTCACATGGCACCGGATTCGA

>Unigene13933_Quanjin

NNNNNANTGANATCTTCTTGGTCTCTGTCAATTTCTTAAGCTCTCCGAGCTGTCAGCACGATACTGTCTGCTAACGTGATAGAGAGATGGCCGTCTCTACAACGACGACAAAATGGTCTCAAACTGTTGTCGGGGGATTTCTCCTCGGAAACGCTGGTGGTCAGTTGGACTGACCGTTAACCGCTTTGAACTGACACTGGCAGAGCTGATTGGAAAACATATGCAGCCGTCTAAGGATACCAAGGCCATACACATGTAGATATAGTTCACATGGCACCGGATTCGA

>Unigene13933_Wujin

TNANAAATCAGGAGATCTTCTTGGTCTCTGTCAATTTCTTAAGCTCTCCGAGCTGTCAGCACGATACTGTCTGCTAACGTGATAGAGAGATGGCCGTCTCTACAACGACGACAAAATGGTCTCAAACTGTTGTCGGGGGATTTCTCCTCGGAAACGCTGGTGGTCAGTTGGACTGACCGTTAACCGCTTTGAACTGACACTGGCAGAGCTGATTGGAAAACATATGCAGCCGCCTAAGGATACGAAGGCCATACACATGTAGATATAGTTCACATGGCACCGGATTCGA

>Unigene12290_Quanjin

NGANANCTCCATTCGTCTACGCGCAGTAACTCGACTTCGGCCTTTGCGCCTACTGCGCCGGATAGGGGGCGTGTTGAGTCCCTGCTGGCTGCTGGAGCGAAAAAGAGTGGGGACGGAGAGGGAGAGGGAGAGTTGACGCTGGCGGATCTGGCGCGGGTGCGCGCGGAACGCGATAGAGAGACCATCGCGGCGGGGCGGCCGCTGTCGGGCCTGCACGCGCGCATCGCGCGCGGCGAGGTCGCGCTCGCCTTTGCGCTCTTCGCGCGTCCGCGTGCCAGCGTGAGCGATGGCAGCGCGAACTCGACTTCGCACAGCAA

>Unigene3662_Quanjin

NNNGTNGNANATACCGCTCGCCAGCATTCCTACCTGATCGCACAGTGGCCGCGACATCTCGTTCTGGACGTCCGTGCAGGCGCTGCGCGACGTGTACAACCTCTCGCTGCTGCTGGCAGCGGTGCTCACGCTCGCCGGCTGCCTCCTCCGCGGCGGCCGCGGCTTCCGCCTCAGTTACAGCAGCCCGGCTGGTTCTACTCGACGACCGCCGGTGTCACCCTGAAATAAAGCAGCCCAGTTTGTACTACTCGAAGACCGCCGGTGTCCCCGTGAGCTTTTTCTTTTTTCTTTTT

>Unigene3662_Banjin

NNANNNGNNNTCNCGCGCTTCGCCACCATTTCCTACCTCCCGCACAGCGGCCGCGACATCTCGTTCTGGACGTCCGTGCAGGCGCTGCGCGACGTGTACAACCTCTCGCTGCTGCTCGCCGCGGTGCTCACGCTCGCCGGCTGCCTCCTCCGCGGCGGCCGCGGCTTCCGCCTCAGTTACAGCTGGCCGGCTGCCTCCTCCGCGGCGGCCGCGGCTTCCGCCTCAGTTACAGCCAT

>Unigene3662_Wujin

GAAGNGNNNNACGCGCTCGCCACCATTCCTACCTGCCGCACAGTGGCCGCGACATCTCGTTCTGGACGTCCGTGCAGGCGCTGCGCGACGTGTACAACCTCTCGCTGCTGCTCGCCGCGGTGCTCACGCTCGCCGGCTGCCTCCTCCGCGGCGGCCGCGGCTTCCGCCTCAGTTACAGC
